# On the psychometric properties and genomic etiology of the general factor of psychopathology

**DOI:** 10.1038/s41380-025-03151-5

**Published:** 2025-08-14

**Authors:** Erik Pettersson

**Affiliations:** Dept of Medical Epidemiology and Biostatistics, Karolinska Institutet, Box 281, 171 77 Stockholm, Sweden

**Keywords:** Psychiatric disorders, Psychology, Genetics

## Abstract

There is a high degree of comorbidity in psychiatry. This implies that there might be a set of underlying common factors that influence multiple conditions. A decade ago, researchers suggested that one way to estimate these is by applying hierarchical factor models, which partition that which is shared among all psychiatric conditions (commonly labeled general psychopathology) from that which is shared by subsets of conditions (commonly labeled specific factors). Whereas the specific factors were relatively uncontroversial, the general factor has been the subject of debate. On the one hand, the general factor appears to predict clinically relevant outcomes; on the other hand, critics have questioned its psychometric foundations and genomic etiolology. In this review, I present evidence that the general factor appears to have sound psychometric properties, and that it is at least possible that it could have a genomic etiology. With the caveat that it is very difficult to identify the data generating processes underlying multivariate data, I end by offering a cautious amendment to the suggestion that the general factor might measure impulsive reactions to emotions.

Comorbidity, or the presence of two or more co-occurring psychiatric conditions, is common in the mental health domain. Roughly half of all individuals who meet diagnostic criteria for one psychiatric diagnosis also meet criteria for additional psychiatric diagnoses [1]. The overlap among psychiatric conditions appears high, regardless of whether based on diagnoses assigned following contact with the mental health system, self- or parent-reported syndromes, or diagnostic interviews in the community [2–6]. Furthermore, the associations between psychiatric conditions and later clinically relevant outcomes often attenuates substantially when co-occurring conditions are statistically adjusted [7, 8], implying that comorbidity in and of itself might be an important predictor of future functioning.

Despite the prevalence and importance of comorbidity, diagnostic manuals primarily focus on specific psychiatric conditions. However, one can use factor analysis, which models a larger number of observed indicators (e.g., psychiatric symptoms, syndromes, or diagnoses) as a function of a smaller number of unobserved (i.e., latent) normally distributed factors, to capture psychiatric comorbidity. A century ago, researchers applied factor analysis to show that intelligence can be conceptualized as existing in a hierarchy, with general intelligence at the top and more narrow substrates below (e.g., working memory, processing speed, etc.) [9, 10]. Likewise, a decade ago, researchers showed that mental health problems can be described by the same hierarchical model with general psychopathology at the top and more specific substrates below (e.g., internalizing, externalizing, and psychotic problems, etc.) [11–13]. Whereas the specific substrates had been well-documented for decades [14, 15], the idea to formally model the top part of the hierarchy (i.e., general psychopathology) was novel.

Research of the past decade has found that the general psychopathology factor predicts future relevant outcomes over and above variation attributable to specific factors (e.g., internalizing and externalizing conditions) [16–21]. For instance, using Swedish twin and population datasets, we observed that the general factor uniquely predicted later clinically relevant outcomes such as suicidal ideation, criminal convictions, and overdoses [7, 22, 23]. and that the magnitude of the associations rivaled those between general intelligence and later educational achievement [5]. As the reporter differed between time 1 and time 2, these longitudinal associations likely cannot be attributed to potential rater biases. This implies that clinicians might benefit from focusing on total symptom load, in addition to specific syndromes or diagnoses, when predicting patient prognosis.

## Critiques of the general factor of psychopathology

Despite its apparent predictive validity, the general psychopathology factor has been critiqued. Researchers have questioned the psychometric properties of the general factor, arguing that a more parsimonious sum score is preferable over a complex latent general factor; that the loadings on the general factor and its associations with outcomes do not replicate; and that the general factor might capture idiosyncratic rater effects [24–26]. Furthermore, researchers have suggested that the general psychopathology factor lacks genomic correlates [27]. Although these critiques are informative and interesting, I suggest that they might be misguided. The goal of this paper is to review and present evidence that the general factor of psychopathology appears to have sound psychometric properties, and that it is at least possible that it could have a genomic etiology.

## On the psychometric properties of the general psychopathology factor

### On the high association between the latent general factor and a total sum score

Fried and colleagues analyzed two waves of psychiatric data from a large epidemiological survey and observed that the correlation between a latent general factor and the corresponding sum score (i.e., sum of all diagnoses) equaled *r* = 0.99 at the first measurement occasion, and *r* = 0.87 at the second measurement occasion. They concluded that because these correlations were so high, it might be wiser to use a simpler sum score that makes no assumptions, rather than a complex latent variable model that makes many assumptions [26].

**Fig. 1:**
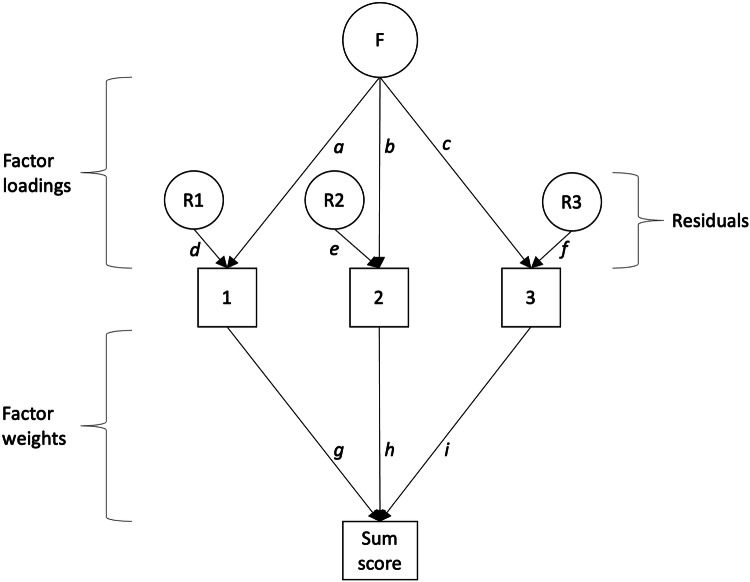
Graphical representation of reliability estimation. Squares 1, 2 and 3 represent observed items, and the square labeled “sum score” represents an observed (weighted) sum score of these three items. F represents a latent factor, and R1, R2, and R3 represent residuals that are not explained by F. Using structural equation modeling tracing rules (assuming unit factor and residual variances), one can estimate the variance explained in the sum score by F (Γ) as a product of the factor loadings (a, b, c) and factor weights (g, h, i): $\Gamma ={{{{\rm{ga}}}}}^{2}+{{{{\rm{hb}}}}}^{2}+{{{{\rm{ic}}}}}^{2}+{{{\rm{gabh}}}}* 2+{{{\rm{gaci}}}}* 2+{{{\rm{hbci}}}}* 2$. One can also estimate the variance in the sum score not explained by F (Θ) as the product of the factor weights (g, h, i) and the residuals (d, e, f): $\Theta ={{{{\rm{gd}}}}}^{2}+{{{{\rm{he}}}}}^{2}+{{{{\rm{if}}}}}^{2}$. Reliability can then be estimated as the variance explained in the sum score by F divided by the total variance: $\Gamma /(\Gamma +\Theta )$. Reliability is typically labeled *alpha* when the factor loadings (denoted *a*, *b*, and *c*) and factor weights (denoted *g*, *h*, and *i*) are constrained to equality, *omega* when the factor loadings are free to vary but the factor weights are constrained to equality, and *determinacy* when both the factor loadings and the factor weights are free to vary.

Although the authors did not frame it as such, the association between a latent factor and its corresponding observed sum score is the definition of several indices of reliability. Briefly, according to test theory, reliability can be estimated as the variance explained in a sum score by its corresponding latent construct [28, 29], depicted graphically in Fig. 1. Unreliability is problematic because it inflates the variance in observed sum scores, which in turn leads to underestimated regression betas and increased measurement error about individuals’ scores. Therefore, from the perspective of reliability theory, a high association between a latent factor and its corresponding sum score is typically considered a positive attribute, as it allows for estimating unbiased regression parameters and individual scores with high precision.

Regardless of whether a high reliability should be considered a vice or virtue, three additional and more technical notes are worth mentioned regarding Fried and colleagues’ critique [26]. First, reliability is usually expressed in terms of variance explained, rather than as a correlation, because variance is used when estimating measurement error about an individual’s score, and how much regression betas might be underestimated by. Squaring the observed correlations renders reliability estimates of 99% and 75% at waves 1 and 2, respectively, of which at least the latter seems less supportive of the original authors’ argument.

Second, Fried and colleagues estimated a latent hierarchical factor model, and, in an unorthodox fashion, correlated the latent factors with the total sum score in the same model [26]. This approach is seldom, if ever, used because sum scores are a perfect linear combination of its indicators such that matrix inversion (a necessary feature of factor analysis) becomes impossible. Although the authors circumvented this issue by estimating the factor model based on the tetrachoric (rather than the Pearson) correlations, I would instead advocate in favor of using standard psychometric techniques when estimating the association between latent factors and their constituent sum scores [30].

Third, irrespective of the suitability of their approach, for multidimensional models, it is important to estimate the associations between all latent factors and a covariate (e.g., a sum score) simultaneously. If one were to constrain the covariate to only be correlated with one of the latent factors in a multidimensional model, that correlation is likely over-estimated. As Fried and colleagues constrained the correlations between the sum score and the three specific factors at zero, the correlation between the general factor and the sum score was likely over-estimated. In the Supplementary materials, I outline this issue algebraically, via simulation, and empirically in a large Swedish sample with information on 9 register-based diagnoses. In brief, alike Fried and colleagues, the correlation between the latent general factor and the total sum score (i.e., sum of 9 diagnoses) was high (*r* = 0.74) when the correlations with the specific factors were fixed at zero. However, the corresponding correlation between the general factor and the total sum score attenuated (*r* = 0.57) when both the general and specific factors were allowed to correlate freely with the total sum score. Thus, the correlation between the latent general factor and its sum score presented by Fried and colleagues was likely overestimated due to imposing unreasonable model restrictions.

At a more abstract level, although their argument about using an (assumption-free) sum score is interesting and mimics past discussions about the lack of merit of latent variable models [31, 32], if one assumes that there is measurement error, then the (complex) latent variable model seems suitable. The choice between these two approaches can play an outsize role when sum scores straddle cutoffs. For example, in a court case where capital punishment hung in the balance, a defendant’s IQ score was just above the cut-off for intellectual disability, such that he was eligible for the death penalty. However, the judges reasoned that it could have been overestimated due to measurement error, and sentenced him to prison [33]. Had the judges instead taken his sum score at face value, the defendant would have been executed.

### On the unreplicability of general factor loadings and its associations with covariates

#### Unreplicability between samples

Researchers have also argued that the associations between the latent general factor and its indicators (commonly labeled factor loadings, as outlined in Fig. 1) do not replicate between samples [24, 25]. For instance, Levin-Aspenson and colleagues analyzed the similarity of the general factor loading patterns across two epidemiological community surveys (*N*s = 8098 and 19,823) and one outpatient clinical sample (*N* = 2900) where the same psychiatric diagnoses were measured [34]. They examined factor similarities via the factor congruence coefficient, which ranges from −1 to 1, with values above 0.95 indicating that two factors are perceived as highly similar [35]. Additionally, because the congruence coefficient can overestimate similarity when factors share the same sign, they also examined if the same factor loadings were equal to or greater than 0.40 (labeled saliency) across samples, and correlations between pairs of factors and their associated statistical significance tests. Levin-Aspenson and colleagues observed high congruences (>0.95) between the two epidemiological samples, but weaker congruence between the epidemiological samples and the outpatient sample. Many indicators from the clinical sample failed the saliency index, and the correlations were of moderate magnitude and mostly non-significant [34]. Based on the totality of these metrics, the authors concluded that general psychopathology had limited consistency between samples.

On a similar note, Watts and colleagues examined the similarity in general factor loadings based on 12 published studies that used the Achenbach School-based Empirically Based Assessment (ASEBA) scales for psychopathology. They observed that the loadings varied considerably on three scales considered particularly relevant for general psychopathology (range: 0.17–0.87), and noted that the poor replicability cannot be attributed to measurement or sample characteristics as the ASEBA scales are exclusively used in studies of youth [25]. Further, they suggested that because the loadings on the general factor do not replicate, its association with relevant outcomes also varies substantially between studies. For example, they noted that the correlation between the general factor and negative emotionality varied substantially (range: *r* = 0.13 to *r* = 0.88) across ten studies [25].

It is worth nothing, however, that others have observed a higher degree of general factor loading similarity across samples [36, 37]. For instance, when we examined four birth cohorts (Swedish males who underwent the mandatory conscription evaluation in 1969–1979 [*n* = 402,931], 1980–1990 [*n* = 438,571], 1990–2000 [*n* = 391,713], and 2000–2010 [*n* = 302,563]), the congruences between general factors (estimated from register-based psychiatric indicators) ranged from 0.99 and 1.0, and the correlations from *r* = 0.57 and *r* = 0.95 (mean *r* = 0.80) [38]. Similarly, the congruences between general factors in mothers and fathers (*N* = 1,572,559 pairs), and in siblings (*N* = 580,891 pairs), equaled 1.0 in both samples, and the correlations equaled *r* = 0.97 and *r* = 0.99, respectively [39, 40]. We also examined the degree to which model fit deteriorated when constraining the loadings to be equal (vs. freely estimated) across the cohorts, parents, and siblings, respectively, within a multi-group framework. The models where the loadings were constrained to equality fit nearly as well (differences in the Comparative Fit Index, ΔCFI, <0.001) [38–40]. Based on simulations, Cheung and Rensvold suggested that a ΔCFI < 0.01 was consistent with interpreting two factors as the same [41]. Furthermore, when we estimated the associations between the general factor and two exposures (resting heart rate and general intelligence assessed during the mandatory Swedish conscription evaluation) separately in the four aforementioned birth cohorts, the standardized regression betas between resting heart rate and the general factor equaled −0.021, −0.020, −0.025, and −0.025, respectively, and the corresponding betas between general intelligence and the general factor were −0.177, −0.183, −0.197, and −0.166 [38]. It would be unexpected to observe factor loadings and associations with independently measured covariates of such similar magnitudes across samples, if general psychopathology were an unreplicable construct.

This begs the question as to why some researchers have observed a relatively high degree of general factor replicability, whereas others have not. One answer might be that that the reviewed studies varied in their sampling and modeling techniques.

Regarding Levin-Aspenson and colleagues’ conclusion that general psychopathology had limited consistency between samples [34], three issues might be worthwhile to consider. First, most loadings that failed the saliency index were attributable to the clinical sample. As acknowledged by Levin-Aspenson and colleagues, the clinical sample was stratified on major depression (72% of the participants had a major depression diagnosis, compared to 18% and 16% in the epidemiological samples, respectively), such that it suffered from range restriction. Indeed, in the two epidemiological samples, the mean of the sample correlation matrix (i.e., the mean of the observed correlations between diagnoses 1 and 2, 1 and 3, etc.) equaled *r* = 0.36 and *r* = 0.37, respectively, whereas the mean of the sample correlation matrix in the clinical sample was substantially lower at *r* = 0.18. Thus, it remains inconclusive if the general factor in the clinical sample failed the saliency index due to it being an unreplicable construct, or due to low sample correlations attributable to stratification.

Second, although Levin-Aspenson and colleagues noted that the correlations between general factor loadings were statistically insignificant [34], the *p*-value is monotonically related to the sample size (holding the effect size constant). As there were 11 indicators (i.e., *N* = 11) and that correlations as large as *r* = 0.48 were non-significant (corresponding to a Cohen’s *d* = 1.09), their tests of statistical significance might have been underpowered.

Third, whereas the congruence coefficient can overestimate factor similarity when loadings share the same sign, the correlation coefficient can underestimate similarity when there is range restriction. As general factors often span a relatively limited range (e.g., there are typically no low loadings), correlations might be lower than congruences. In the Supplementary materials, I show that two factors simulated to have a high congruence coefficient became moderately correlated after restricting the loading range to that of typical general factors. In other words, the high congruence coefficients and moderate correlations between the general factors in the two epidemiological samples reported by Levin-Aspenson and colleagues appear to correspond to what one might observe between two highly associated, but range-restricted, factors.

Regarding the 11 studies (one of the 12 studies does not appear to include the three ASEBA scales of interest [42]) where Watts and colleagues noted that general factor loadings varied considerably (range: 0.17–0.87), the 8 lowest loadings (<0.40) stemmed from a single data set, namely, the Generation R study of Dutch children. In the first analysis of this data, the researchers fit a hierarchical factor model *jointly* to parent-ratings at age 6, observer-ratings based on a puppet interview at age 6, and teacher-ratings at age 6 [43]. In a second analysis, they additionally included parent- and self-ratings at age 10 [44]. Six of the eight lowest loadings stemmed from the teacher ratings, and the remaining two from self-rated thought problems at age 10, and (possibly^1^) observer-rated depression based on the puppet interview at age 6. While it is interesting that ratings by teachers at age 6 do not converge with ratings by parents at age 6 and 10, it is questionable if that should be counted against the replicability of the general factor. In other words, the three scales of interest were consistently associated with general psychopathology (loadings ≥ 0.40) across all 11 studies save for the (primarily teacher-based ratings at age 6 in) the Generation R study.

On a similar note, the 10 reviewed studies where the correlations between the general factor and negative emotionality varied substantially (the five lowest correlations were around *r* = 0.20, and two highest correlations were *r* > 0.80) also varied in their sampling and modeling techniques [25]. It is well-known correlations tend to be higher when using cross-sectional data, a single rater, and latent factor modeling (to control measurement error). Regarding the two studies where the correlations were *r* > 0.80, the samples were cross-sectional, the reporter was the same, and measurement error was controlled via latent variable modeling [45, 46]. In contrast, regarding the five studies where the correlations with negative emotionality were around *r* = 0.20, two of those studies were longitudinal (across 2 and 12 years, respectively) and the raters additionally differed between time 1 and 2 in one study [47, 48]. Another of the low correlations was based on 3-year old children where the raters differed (mother-reported general psychopathology, and father-reported negative emotionality) [49]. The last two of the low correlations actually appear to belong among the group of medium-sized correlations: for one of the low correlations, Watts and colleagues reported the unique beta (i.e., regression of the general factor on negative emotionality, adjusted for covariation with other personality traits), rather than the unadjusted beta that was more in line with the medium-sized correlations (standardized *b* = 0.55) [50]. The other low correlation is (potentially^2^) a typo: in the Supplemental materials of the cited study, the correlations between negative emotionality and the general factor ranged between 0.45–0.54 depending on measurement model [24].

Thus, although Watts and colleagues claimed that “measurement and sample characteristics… are clearly not the leading causes of their poor replicability.” (p114/115) [25], the variability in general factor loadings and its associations with negative emotionality in the reviewed studies appear attributable to systematic measurement and sampling designs known to boost versus suppress associations.

#### Unreplicability within samples

Watts and colleagues argued that general factors should be robust to scale exclusion. To test this, in a sample of children (*N* = 2498) rated by their parents on psychiatric scales, they removed one scale at a time in an iterative fashion [24]. They observed that the (absolute) factor congruence coefficients between the general factor based on the original solution that included all scales, and each of the ensuing general factors that omitted one scale at a time, ranged between 0.11 and 1.0 (similarly, the absolute correlations ranged between 0.01 and 0.99), indicating that some general factors, after excluding a scale, were dissimilar to the original solution.

To date, this exclusion test has only been applied in this sample. To examine if similar results were to emerge in other samples, I analyzed 10 datasets (Table 1): the National Comorbidity Survey (NCS); the Collaborative Psychiatric Epidemiology Study (CPES); the Methods to Improve Diagnostic Assessment and Services (MIDAS); the National Comorbidity Study-Replication (NCS-R); the National Epidemiological Survey on Alcohol and Related Conditions (NESARC); Swedish adults with information on 14 psychiatric diagnoses (available in Supplementary Table 1A); and the four aforementioned male birth cohorts with information on 10 psychiatric diagnoses, anti-depressant prescription, and criminal convictions (available in Supplementary Table 2A) [5, 26, 34, 38, 51]. To maintain consistency with the original analyses, for the NCS, CPES, and MIDAS [34], I extracted one exploratory general factor, two exploratory factors from the NCS-R, and four exploratory factors from the male birth cohorts [51]. Lacking a precedent, I extracted three exploratory factors from the NESARC sample (the first five Eigenvalues were 5.42, 1.81, 0.91, 0.56, 0.44), and four factors from the Swedish population sample (the first five Eigenvalues were 6.73, 1.32, 1.21, 1.07, 0.66). For the NESARC, Swedish population sample, and the male birth cohorts, I rotated the original sample (that was based on all indicators) with a bifactor rotation (with orthogonal specific factors), and then target rotated the factor solutions that excluded one indicator at a time toward the original bifactor rotation [52]. For the NCS-R where the authors analyzed 2 factors, the bifactor rotation is not feasible; instead, I rotated the solution toward the principal components (as a general factor proxy; R-code available in the online Supplement). Then, for each of the 10 datasets, I computed the factor congruence and correlation coefficients between the general factor based on the original solution, and a general factor where I had omitted one indicator at a time.

**Table 1:** Factor congruences and correlations between general factors after excluding one indicator at a time across 10 samples.

Sample and study source	Number of factor indicators	Sample size	Number of exploratory factors	Rotation	Mean congruence coefficient^a^ (min, max)	Mean correlation coefficient^a^ (min, max)
NCS [34]	11	8098	1	–	1.00 (1.00, 1.00)	0.98 (0.95, 1.00)
CPES [34]	11	19,823	1	–	1.00 (1.00, 1.00)	0.98 (0.94, 1.00)
MIDAS [34]	11	2900	1	–	0.99 (0.97, 1.00)	0.77 (0.31, 0.94)
NCS-R [34]	15	3199	2	PC	1.00 (1.00, 1.00)	0.99 (0.98, 1.00)
NESARC [26]	11	43,093	3	Bifactor	1.00 (0.99, 1.00)	0.96 (0.90, 1.00)
Swedish population sample [5]	14	909,699	4	Bifactor	1.00 (0.99, 1.00)	0.91 (0.63, 1.00)
Swedish male birth cohort 1 [38]	13	402,931	3	Bifactor	1.00 (0.98, 1.00)	0.97 (0.62, 1.00)
Swedish male birth cohort 2 [38]	13	438,571	3	Bifactor	1.00 (0.99, 1.00)	0.98 (0.88, 1.00)
Swedish male birth cohort 3 [38]	13	391,713	3	Bifactor	1.00 (1.00, 1.00)	1.00 (1.00, 1.00)
Swedish male birth cohort 4 [38]	13	302,563	3	Bifactor	1.00 (1.00, 1.00)	0.99 (0.93, 1.00)

The four Swedish birth cohorts consisted of males who were conscripted between 1969 and 1980, between 1980 and 1990, between 1990 and 2000, and between 2000 and 2010, respectively [38].

*NCS* national comorbidity survey, *CPES* collaborative psychiatric epidemiology surveys, *MIDAS* methods to improve diagnostic assessment and services, *NCS-R* national comorbidity survey-replication, *NESARC* national epidemiologic survey on alcohol and related conditions, *Sweden* all swedish individuals aged 35–45 years old, *PC* principal component.

^a^The factor congruence and correlation coefficients were computed between the original (exploratory) general factor, and a corresponding general factor after excluding one indicator at a time from the hierarchical solutions. These columns display the mean, minimum, and maximum coefficients based on the entire the range of coefficients.

Table 1 shows that the congruence coefficients ranged between 0.97 and 1.00, and the correlation coefficients between 0.31^3^ and 1.0, across all 10 data sets and indicator exclusions. This implies that the loadings on the general factors remained quite similar regardless of which indicator was excluded. Thus, although Watts and colleagues stated that general psychopathology factors are “extremely sensitive to their contents” (pp. 116) [24], at least in these 10 samples, the opposite seemed true.

#### Associations between factor scores based on general factors with different loadings

In any event, it remains uncertain if variability in general factor loadings substantially changes the rank-ordering of individuals on observed general factor scores. To examine this, I analyzed eight samples (sample descriptives are displayed in Table 2). I first fit two types of measurement models (a multivariate confirmatory bifactor model, and a 1-factor model) to these samples, and, second, I re-estimated these models but where I fixed the loadings on the general factors in an inverted order (all models are displayed in Supplementary Tables 1A–9C). For instance, in a sample of individuals with information on 14 psychiatric diagnoses, in the first confirmatory bifactor model, the three highest loadings on the general factor were depression (loading = 0.79), anxiety (loading = 0.77), and ADHD (loading = 0.73), and the three lowest loadings were eating disorders (loading = 0.47), oppositional-defiant disorder (ODD; loading = 0.48), and tics (loading = 0.51). In the second confirmatory bifactor model, where I fixed the loadings on the general factor in an inverted order, the three highest loadings were eating disorders (loading = 0.79), ODD (loading = 0.77), and tics (loading = 0.73), and the three lowest loadings were ADHD (loading = 0.51), anxiety (loading = 0.48), and depression (loading = 0.47). I then extracted and correlated the factor scores from the freely estimated and inverted general factors.

**Table 2:** Correlations between general factor scores based on the freely estimated general factor loading pattern, and a general factor loading pattern fixed in an inverted order.

Sample and study source	Age	Sample size	Rater	Observed data	Confirmatory bifactor model	1-factor model
CATSS twins [88]	9	8748	Parent	62 neuro-developmental, conduct, and anxiety symptoms	*r* = 0.91	*r* = 0.97
CATSS twins [88]	18	4245	Parent	74 ABCL symptoms	*r* = 0.87	*r* = 0.97
STAGE twins [23]	20–45	11,716	Self	48 internalizing, externalizing, and neuro-developmental symptoms	*r* = 0.64	*r* = 0.95
Swedish population sample [5]	35–45	909,699	Psychiatrists	14 psychiatric diagnoses	*r* = 0.93	*r* = 0.98
Swedish male birth cohort 1 [38]	51–64	402,931	Psychiatrists; courts	10 psychiatric diagnoses, prescribed anti-depressants, and criminal convictions	*r* = 0.80	*r* = 0.93
Swedish male birth cohort 2 [38]	42–62	438,571	Psychiatrists; courts	10 psychiatric diagnoses, prescribed anti-depressants, and criminal convictions	*r* = 0.82	*r* = 0.90
Swedish male birth cohort 3 [38]	33–52	391,713	Psychiatrists; courts	10 psychiatric diagnoses, prescribed anti-depressants, and criminal convictions	*r* = 0.81	*r* = 0.91
Swedish male birth cohort 4 [38]	23–40	302,563	Psychiatrists; courts	10 psychiatric diagnoses, prescribed anti-depressants, and criminal convictions	*r* = 0.70	*r* = 0.90

The models are displayed in Supplementary Tables 1A–9C.

*ABCL* adult behavior checklist, *CATSS* the child and adolescent twin study in sweden.

The four Swedish birth cohorts consisted of males who were conscripted between 1969 and 1980, between 1980 and 1990, between 1990 and 2000, and between 2000 and 2010, respectively.

Across the eight samples and two measurement models, as shown in Table 2, the mean correlation between the original and the inverted general factor scores equaled *r* = 0.87 (range: 0.64, 0.98). Thus, even when the *loadings* on the general factors (by design) correlated at *r* = −1.00, the corresponding *observed factor scores* remained strongly positively associated [36, 37, 53]. One possibility might be that general factors are not only defined by the relative ordering of their indicators, but also by all indicators that load substantially on them.

### On the general factor and idiosyncratic rater effects

Another concern with hierarchical psychopathology models is whether the general factor captures true trait variance, versus idiosyncratic rater biases. If ratings were wholly attributable to rater biases, then the correlation between different reporters ought to be close to the null. Research, however, indicates that the general factor is moderately correlated between reporters [42, 54–56]. For instance, in the test manual of the Child Behavior Checklist, the mean of the correlations among self-, parent-, and teacher-ratings on the total problems scale (a general factor proxy) equaled *r* = 0.54 (range depending on rater pairs: 0.21, 0.80) [57]. Although this might be partly attributable to shared biases (e.g., parents might reach a shared conclusion after discussing their children’s characteristics), ratings by independent observers also seem to converge. Across five samples, adults who self-reported high on the first principal component of an omnibus psychopathology questionnaire (i.e., the Minnesota Multiphasic Personality Inventory) were rated by independent observers as displaying significantly more traits related to distress and impairment (e.g., they were rated as more likely to “be cold and distant in relationships with others”, “feel guilty”, and “feel a lack of personal meaning in life”) [58].

These studies, however, did not examine whether the magnitude of the general factor decreased after adjusting for idiosyncratic rater effects. Watts and colleagues examined this issue in three samples of children that included self-, parent-, and teacher-ratings on the same 18 internalizing and externalizing symptoms (*N*s ranged from 303–2119) [59]. They observed that the magnitude of the general factor attenuated after adjusting for rater effects, and concluded that it remains uncertain whether this might be attributable to idiosyncratic rater biases, or to whether the raters observed targets in different contexts. To address this, it would be necessary to analyze a sample in which targets are observed by multiple raters in the same context.

Such a sample is available from the Institute of Personality and Social Research (IPSR) that included University of California students and community members (*N* = 940) who participated in an in-depth study of personality [60]. Between 5–8 judges (ISPR staff and psychology graduate students) observed the participants over 1–3 days while they engaged in a series of structured (e.g., interviews and leaderless discussions) and unstructured contexts (e.g., during meals and coffee breaks). At the end, the judges rated the participants on 100 broad-based personality descriptors. To isolate idiosyncratic rater effects, the ratings on the personality descriptors were averaged across all judges.

In subsequent exploratory factor analyses of these mean ratings [60], the factors were (Varimax) rotated to improve interpretability, which tends to obscure a potential general factor. I therefore (un)rotated the factor solution toward the first principal component (PC; R-code in online supplement). As displayed in Table 3, the first PC of (the mean across raters on) these personality ratings appeared to contrast negative (“anxious” = 0.76; “distrustful” = 0.56; and “emotionally bland” = 0.45) versus positive impressions (e.g., “social poise, presence” = −0.80; “aware of own motives” = −0.48; and “productive” = −0.39). Thus, when multiple raters simultaneously observed targets in the same context, a dimension akin to a general factor still emerged. Coupled with the observation that general factors predict future clinically relevant outcomes even when the raters differ [7, 17, 22, 23, 61], one possibility might be that the general factor is not solely attributable to idiosyncratic rater effects.

**Table 3:** Loadings of 100 observer-rated personality items on the first principal component (PC1).

Item	PC1 loading
Fearful	0.77
Brittle ego defenses	0.76
Anxious	0.76
Self-defeating	0.75
Self-pitying	0.73
Self-defensive	0.65
Ruminates	0.64
Concern with own adequacy	0.63
Sensitive to criticism	0.61
Feels lack of meaning	0.61
Avoids relationships	0.60
Troubled by uncertainty	0.58
Gives up when frustrated	0.57
Distrustful	0.56
Expresses anxiety bodily	0.54
Delays, avoids action	0.54
Hostile	0.51
Subtly negativistic	0.46
Projects	0.46
Irritable	0.45
Repressive, dissociative	0.45
Emotionally bland	0.45
Guilt-prone	0.44
Sensitive to demands	0.44
Over-control	0.42
Fluctuating moods	0.42
Sees things complexly	0.41
Does not vary roles	0.40
Compares self to others	0.36
Submissive	0.35
Physiological concern	0.35
Seeks reassurance	0.32
Fantasizes	0.30
Moralistic	0.29
Has conservative values	0.27
Introspective	0.25
Extrapunitive	0.25
Unpredictable	0.17
Judges conventionally	0.16
Fastidious	0.12
Guileful, manipulative	0.09
Critical, skeptical	0.08
Condescending	0.08
Creates dependency	0.06
Nonverbal communication	0.04
Sees self as “objective”	0.03
Arouses nurturance	0.02
Protective	0.00
Rebellious	−0.02
Ethically consistent	−0.03
Thinks unconventionally	−0.04
Assesses others’ motives	−0.05
Concern with deep issues	−0.05
Self-indulgent	−0.06
Direct in hostility	−0.09
Cannot wait for reward	−0.10
Stretches limits	−0.13
Masculinity-femininity	−0.14
Dependable, responsible	−0.15
Aesthetically reactive	−0.16
Eroticizes situations	−0.16
Power oriented	−0.16
Values own autonomy	−0.18
Values intellect	−0.21
Self-dramatizing	−0.21
Sympathetic	−0.22
Giving	−0.24
Sensuous	−0.26
Heterosexual interest	−0.28
Aspiration level	−0.29
Proffers advice	−0.29
Sees self as attractive	−0.31
Internally consistent	−0.34
Calm, relaxed in manner	−0.35
Aware of impression made	−0.37
Physically attractive	−0.38
Intellectually capable	−0.39
Productive	−0.39
Candid	−0.39
Unaware of self-concern	−0.40
Rapid tempo	−0.44
Responds to humor	−0.45
Warm, compassionate	−0.46
Gregarious	−0.46
Wide interests	−0.48
Aware of own motives	−0.48
Sees heart of problems	−0.48
Assertive	−0.50
Nonverbally expressive	−0.51
Talkative	−0.52
Turned to for advice	−0.55
Arouses liking	−0.56
Cheerful	−0.56
Socially perceptive	−0.58
Initiates humor	−0.59
Verbally fluent	−0.61
Interesting	−0.63
Skillful in social play	−0.64
Charming	−0.72
Social poise, presence	−0.80

The original solution was presented by Lanning [60], which I in turn (un)rotated toward the first principal component (see text).

## On the genomic etiology of the general factor: the Q_SNP_ comparison

Another challenge leveled at the general factor of psychopathology is that it lacks genomic correlates. Specifically, Grotzinger and colleagues developed two editions of the so-called Q_SNP_ comparison, of which the second edition failed to support that SNPs are associated with general psychopathology [27]. This conclusion stands in contrast to family studies that have shown that the high degree of genetic overlap among psychiatric conditions appears partly attributable to general psychopathology [18, 36, 62–64]. Additionally, by fitting separate hierarchical models to genetic and environmental covariance matrices, Lahey and colleagues observed that whereas internalizing and externalizing factors emerged in both the genetic and environmental covariance matrices, general psychopathology emerged primarily in the genetic covariance matrix [65], an observation we replicated in Swedish twin children and adult siblings [66, 67]. Aside from family designs, studies of single nucleotide polymorphisms (SNPs) have also highlighted that the general factor has a partly genetic origin [63, 68].

The first edition of the Q_SNP_ comparison converged with these results. In this version, the Q_SNP_ involved first estimating the association between a SNP and the latent general factor, and then, in a second model, examine if SNPs were directly associated with factor indicators above and beyond that accounted for by the pathway via the general factor [69]. When applying this Q_SNP_ comparison to a uni-dimensional general factor model based on five psychiatric diagnoses (capturing psychotic and internalizing problems), Grotzinger and colleagues identified 128 SNPs that were associated with the latent general factor (i.e., where the additional direct paths from SNPs to the observed indicators added no information) [69].

However, in a follow-up study that fit multi-dimensional hierarchical factor models to eleven psychiatric diagnoses (capturing psychotic, internalizing, externalizing, and neurodevelopmental conditions), and using an updated version of the Q_SNP_ comparison (described below), Grotzinger and colleagues observed a mixed pattern of results [27]. Whereas the Q_SNP_ comparison identified 66 SNPs that were associated with a bifactor general factor, a higher-order general factor was only associated with 2 SNPs. Grotzinger and colleagues concluded that there was “little utility of a single dimension of genetic risk across psychiatric disorders“ (pp. 548) [27].

As this conclusion rests on the Q_SNP_ comparison, this test is worth a more detailed look. As noted above, the Q_SNP_ comparison examines whether a simpler model can account for the data as well as a more complex model, and if so, then the complex model is rejected based on parsimony. In its second edition, the Q_SNP_ comparison varies by type of hierarchical model. For higher-order models, SNPs are *only* associated with the general factor in the simple model, whereas SNPs are *only* associated with the specific factors in the complex model. For the bifactor model, SNPs are *only* associated with the general factor in the simple model, whereas SNPs are associated with *both* the general and the specific factors in the complex model. For both the higher-order and bifactor models, when the simple model fit significantly worse than the complex one (i.e., when the simple model could not account for the data as well as the complex model), Grotzinger and colleagues concluded that SNPs were not associated with the general factor [27].

Although the Q_SNP_ comparison might perform well when the data generating process matches either the simple or complex models, it might be biased if polymorphisms were to influence *both* general and specific psychopathology factors. To that end, I simulated two populations with factor structures matching the higher-order and bifactor model parameters reported by Grotzinger and colleagues [27], respectively, and added a covariate (akin to a SNP) in several simulations.

In the first simulation, the covariate was only associated with the general factor. In a second set of simulations, the covariate was associated with *both* the general and one (or more) specific factor(s). The standardized beta equaled 0.05 in all simulations. I then sampled (with *N* = 10,000) from these simulated populations 100 times [70, 71], and applied the higher-order and bifactor Q_SNP_ comparisons to examine how often they identified that the covariate was associated with the general factor in the simulated population (i.e., its statistical power). In the second set where the covariate was simulated to be associated with both the general and one (or more) specific factors, the Q_SNP_ comparison is bound to be wrong, but these simulations reveal whether the Q_SNP_ comparison tends to be wrong more in one direction than the other.

The results are summarized in Table 4. The first row shows that the Q_SNP_ comparison performed well when the covariate was simulated to *only* be associated with the general factor: it correctly rejected the more complex model in favor of the simpler one, on average, 96% of the time (i.e., it had 96% power to detect the association between the SNP and the general factor).

**Table 4:** Power simulation of hierarchical factor analytic models regressed on a covariate.

	Power of Q_SNP_ comparison to detect regression of general factor (p) on covariate^a^		Power to detect regression of general factor (p) on covariate
Simulated population model: Regressions of general (p) and specific factors (S) on covariate^b^	Sample model: Bifactor model Q_SNP_ comparison	Sample model: Higher-order model Q_SNP_ comparison	Sample model: Covariate - > bifactor model (*p*, S1, S2, S3, S4)
Covariate - > *p*	97%	95%	92%
Covariate - > *p*, S1	5%	4%	93%
Covariate - > *p*, S2	9%	2%	95%
Covariate - > *p*, S3	2%	0%	92%
Covariate - > *p*, S4	20%	1%	92%
Covariate - > *p*, S1, S2	0%	0%	94%
Covariate - > *p*, S1, S3	0%	1%	94%
Covariate - > *p*, S1, S4	0%	1%	92%
Covariate - > *p*, S2, S3	1%	0%	94%
Covariate - > *p*, S2, S4	2%	2%	93%
Covariate - > *p*, S3, S4	0%	3%	92%
Covariate - > *p*, S1, S2, S3	0%	0%	94%
Covariate - > *p*, S1, S2, S3, S4	0%	–^c^	92%

The measurement models were always matched for the simulated population and the sample (e.g., when the simulated population measurement model corresponded to a bifactor model, so did the sample model). A total of 100 samples (*N* = 10,000) were drawn from the population, and the population regression beta equaled 0.05 for all simulations. See text for further simulation and Q_SNP_ comparison details. p means general factor; S1 (specific factor 1) captured primarily obsessive-compulsive disorder and anorexia; S2 captured schizophrenia and bipolar disorder; S3 captured primarily ADHD, autism, and post-traumatic stress disorder; and S4 captured primarily anxiety and depression. The full set of factor loadings are available in the original publication [27].

^a^For row 1, the percentage captures how often the Q_SNP_ comparison correctly identified the simulated association between the covariate and the general factor (i.e., its statistical power). For the remaining rows, the percentage represents how often the Q_SNP_ comparison rejected the simpler model (where the covariate was only associated with the general factor) in favor of the more complex model (where the covariate was primarily associated with the specific factors). For these rows, given that the covariate was simulated to be associated with both the general and one (or more) specific factors, a low percentage indicates that the Q_SNP_ comparison is biased toward incorrectly rejecting the utility of the general factor.

^b^This column displays the simulated population. For instance, “Covariate - > p, S1” means that the general factor and specific factor 1 were associated with the covariate in the simulated population (and that the covariate was not associated with specific factors 2–4).

^c^This model cannot be estimated because it lacks one degree of freedom [61].

However, rows 2–13 in Table 4 display that the Q_SNP_ comparison consistently rejected the simpler model in favor of the more complex model when the covariate was simulated to be associated with both the general and one (or more) specific factor(s). Specifically, the Q_SNP_ comparison rejected the utility of the general factor, on average, close to 98% of the time, despite that the covariate and general factor were associated in the simulated populations. Similar results emerged in an additional simulation when the general factor beta equaled 0.08 and the specific factor beta(s) equaled 0.04 (Supplementary Table 10), with the Q_SNP_ comparison erroneously rejecting the utility of the general factor more than 92% of the time even when the association was twice as large with the general compared to the specific factor(s). Although it is impossible to know whether the simulated population structure matches the true data generating process, given the pleiotropic nature of genes, it might at least be conceivable that SNPs could influence both general and one (or more) specific psychopathology factors.

Rather than relying on the Q_SNP_ comparison, which appears biased towards erroneously rejecting the utility of the general factor under the second set of simulations, an alternate approach is to simultaneously regress all factors in (bifactor) hierarchical models onto each SNP. As displayed in the last column of Table 4, this approach performed about as well as the Q_SNP_ comparison when the covariate was simulated to *only* be associated with the general factor (mean power to detect the association between the covariate and the general factor = 92%). Furthermore, and in contrast to the Q_SNP_ comparison, it also performed well when the covariate was simulated to be associated with both the general and one (or more) specific factor(s) (mean power to detect the association between the covariate and the general factor = 93%). As this simultaneous regression approach performed well regardless of the data generating process, it might be preferable over the Q_SNP_ comparison when examining associations between SNPs and (bifactor) hierarchical models.

## What might general psychopathology measure?

Although I have argued that the general factor of psychopathology appears to have sound psychometric properties and at least a potential genomic etiology, another challenge is that data-generating mechanisms without a general factor can still produce observed data that allow for the extraction of a general factor [13, 72–75]. This challenge, which plagues all multivariate domains including for example intellectual abilities, implies that the general factor is difficult to falsify. For instance, an alternate speculation is that the general factor could be an outcome, rather than a cause, of more specific forms of psychopathology [25].

Although plausible, observed data does not appear to fit such a data generating mechanism. Specifically, it has been shown that if two latent factors (e.g., internalizing and externalizing problems) were to cause one another over time, the data eventually becomes uni-dimensional [76]. Thus, if this were the data generating process, then one would expect the magnitude of the general factor to increase over time. However, a meta-analysis of 65 longitudinal studies found that the magnitude of general psychopathology remained constant from childhood to adolescence [77]. Additionally, when I examined two other data generating mechanisms that would follow if the general factor were an outcome, the observed data did not match [78]. Nevertheless, it is probably wise to interpret such findings cautiously, given the challenge of inferring the data generating process underlying multivariate data.

With this caveat in mind, if the general factor of psychopathology purportedly were to quantify a trait-like process (when it is typically labeled p), there are several suggestions for what it might measure, including distress and impairment, psychotic or irrational thinking, and dispositional impulsivity [13, 79, 80]. Of these, currently, at least some empirical studies lean toward favoring the role of impulsivity [45, 81], an idea proposed by Carver and colleagues [80]. Prior to speculating on the origins of the general factor, Carver argued that the function of affect was to alert organisms as to whether they were moving toward or away from rewarding and threatening stimuli, respectively, at adequate pace [82]. He later supplemented this theory by suggesting that individuals additionally vary in how sensitive they are toward affect as a whole, which he argued was captured by trait impulsivity [83]. Drawing on this model, Carver and colleagues proposed that immediate responses to emotions without reflective delay are invariably associated with adverse outcomes, such that impulsivity might be correlated with the variance that is shared by all symptoms [80].

The perspective that impulsivity is important for broad mental health also has historical antecedents. Inspired by psychoanalytic theory and developmental psychology, in the 1950s, Block coined the concept *ego-control*, which he suggested captured permeability of perceptions and impulses [84]. He noted that although children develop ego-control as they grow up – in contrast to Carver and colleagues [80] – Block argued that this process could go too far, that is, that too much impulse control could become problematic and that either extreme on this continuum is associated with both adaptive and maladaptive behaviors: “…overcontrol may lead to personal immobilization and adaptive rigidity. And undercontrol, if not too extreme, can have adaptive implications by contributing to spontaneity and warmth, creativity, and the seizing of opportunities lost if unclaimed” (pp. 179) [85].

Block further suggested that *ego-resiliency* captured the ability to adjust one’s level of ego-control to meet fluctuating situational demands, and that maximal ego-resiliency entailed being “as undercontrolled as possible and as overcontrolled as necessary” (pp. 351) [86]. A re-analysis of Block’s rich longitudinal study (*N* = 157) found that (observer-rated) ego-resiliency was strongly inversely correlated with a (self-rated) general factor of personality [87]. One cautious speculation might be that the variance that is shared by all symptoms is not necessarily associated with impulsivity per se, as advocated by Carver and colleagues [80], but rather with the struggle of calibrating self-control to match situational demands. More broadly, perhaps individuals who struggle to temporarily change their typical way of behaving or thinking according to fluctuating circumstances might in turn be at increased risk of virtually all psychiatric phenomena.

## Conclusion

Since its inception a decade ago, the general psychopathology factor has been a source of contention. I suggest that some of the critiques leveled against the psychometric properties and etiology of the general factor might be misguided. An additional critique is that it is difficult to rule out data generating processes that lack a general factor, a complexity shared by other multivariate constructs such as general intelligence. Nevertheless, even though the data generating process underlying intellectual abilities remains murky, general intelligence has arguably been useful for predicting school and work performance and for diagnosing intellectual disabilities. Similarly, the general factor of psychopathology might help researchers identify transdiagnostic risk factors and clinicians predict patient prognosis.

## Supplementary information

Supplemental materials

## Footnotes

1 Professor Watts did not respond to requests for clarification.

2 Professor Watts did not respond to requests for clarification.

3 The clinical sample (MIDAS) generated notably lower correlations than the other samples, perhaps because it was stratified on major depression, as noted above.
