# Supplementary material for: On the psychometric properties and genomic etiology of the general factor of psychopathology
Source: Mol Psychiatry. 2025 Aug 14;30(11):5523–33. doi: 10.1038/s41380-025-03151-5 (PMC12532720; doi:10.1038/s41380-025-03151-5)
Supplement: Supplementary file 1 — Supplemental materials [file 41380_2025_3151_MOESM1_ESM.docx]

**Supplementary Materials**

**Supplement 1.** On the peril of associating a covariate with only one factor in a multidimensional factor analysis model with cross-loadings

**Supplement 2.** On the effect of range restriction on the factor congruence and correlation coefficients.

**Supplement 3.** R-code for rotating a factor analytic solution toward principal components

**Supplementary Table 1A.** Confirmatory bifactor factor analyses of 14 psychiatric diagnoses in Swedish population sample (age 35-45).

**Supplementary Table 1B.** 1-factor models of 14 psychiatric diagnoses in Swedish population sample (age 35-45).

**Supplementary Table 2A.** Confirmatory bifactor models of psychiatric conditions, prescribed anti-depressants, and crimes in Swedish male birth cohort 1 (age 51-62).

**Supplementary Table 2B.** 1-factor models of psychiatric conditions, prescribed anti-depressants, and crimes in Swedish male birth cohort 1 (age 51-62).

**Supplementary Table 3A.** Confirmatory bifactor models of psychiatric conditions, prescribed anti-depressants, and crimes in Swedish male birth cohort 2 (age 42-62).

**Supplementary Table 3B.** 1-factor models of psychiatric conditions, prescribed anti-depressants, and crimes in Swedish male birth cohort 2 (age 42-62).

**Supplementary Table 4A.** Confirmatory bifactor models of psychiatric conditions, prescribed anti-depressants, and crimes in Swedish male birth cohort 3 (age 33-52).

**Supplementary Table 4B.** 1-factor models of psychiatric conditions, prescribed anti-depressants, and crimes in Swedish male birth cohort 3 (age 33-52).

**Supplementary Table 5A.** Confirmatory bifactor models of psychiatric conditions, prescribed anti-depressants, and crimes in Swedish male birth cohort 4 (age 23-40).

**Supplementary Table 5B.** 1-factor models of psychiatric conditions, prescribed anti-depressants, and crimes in Swedish male birth cohort 4 (age 23-40).

**Supplementary Table 6A.** Confirmatory bifactor factor analysis of 62 symptoms in the CATSS sample (age 9).

**Supplementary Table 6B.** Confirmatory bifactor factor analysis of 62 symptoms in the CATSS sample (age 9), after fixing the loadings on the general factor in an inverted order.

**Supplementary Table 7C.** 1-factor model of 62 symptoms in the CATSS sample (age 9) before and after fixing the loadings on the general factor in an inverted order.

**Supplementary Table 8A.** Confirmatory bifactor factor analyses of 74 Adult Behavior Checklist symptoms in the CATSS sample (age 18).

**Supplementary Table 8B.** Confirmatory bifactor factor analysis of 74 Adult Behavior Checklist symptoms in the CATSS sample (age 18), after fixing the loadings on the general factor in an inverted order.

**Supplementary Table 8C.** 1-factor model of 74 Adult Behavior Checklist symptoms in the CATSS sample (age 18), before and after fixing the loadings on the general factor in an inverted order.

**Supplementary Table 9A.** Confirmatory bifactor factor analysis of 48 symptoms in the STAGE sample (age 20-45).

**Supplementary Table 9B.** Confirmatory bifactor factor analysis of 48 symptoms in the STAGE sample (age 20-45), after fixing the loadings on the general factor in an inverted order.

**Supplementary Table 9C.** 1- factor models of 48 symptoms in the STAGE sample (age 20-45) , before and after fixing the loadings on the general factor in an inverted order.

**Supplementary Table 10.** Power simulation of hierarchical factor analytic models regressed on a covariate

**Supplement 1. On the peril of associating a covariate with only one factor in a multidimensional factor analysis model with cross-loadings**

When a covariate is associated with a multidimensional model with cross-loadings, it is important to allow the covariate to be associated with all factors. To the extent the association is constrained at zero with one (or more) of the factors, the remaining and freely estimated associations are potentially over-estimated. Consider the figure below that depicts a bifactor model based on y_k_ observed indicators, from which one general (p) and two specific (S1, S2) latent factors have been extracted, and where there is an observed covariate (X).


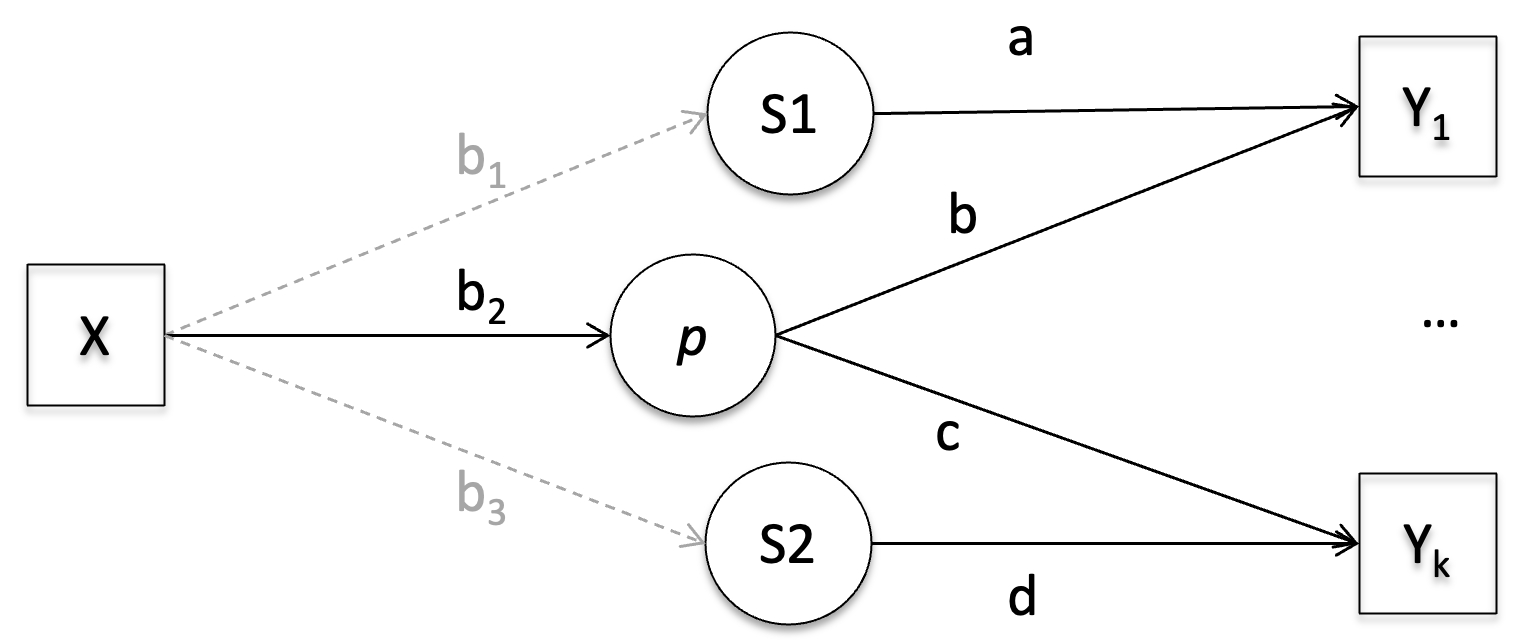


In the above figure, when the covariate (X) is constrained to only be associated with the general factor (i.e., when the paths b_1_ and b_3_ are constrained at zero), then the model-implied correlation between the covariate (X) and the factor indicator (Y_1_) is modeled based on the following paths (assuming standardized data):

r_x.y1_ = b_2_*b

However, when the covariate (X) is allowed to correlate freely with all latent factors (i.e., when X is associated with p, S1, and S2), then the model-implied association between the covariate (X) and the factor indicator (Y_1_) is modeled via the following paths:

r_x.y1_ = b_2_*b + b_1_*a

If the pathway b_1_*a is greater than zero in the population, then the constrained model (where the covariate is only allowed to correlate with the general factor) will likely overestimate the b_2_ parameter.

To demonstrate this via simulations, I created a population where the measurement model corresponded to that reported by Fried and colleagues at the first wave of assessment.^1^ I then regressed the measurement model onto a (standardized) covariate and varied the magnitudes of the betas according to the first column in Table A below. Specifically, in the first simulated population, the covariate was associated with the general factor at beta = 0.80, and the three specific factors at beta = 0.80. In the second simulated population, the covariate was associated with the general factor at beta = 0.80, and the three specific factors at beta = 0.60. In the third simulated population, the covariate was associated with the general factor at beta = 0.80, and the three specific factors at beta = 0.40.

I then sampled 5,000 individuals from these three populations 100 times, and fit two models to these samples. In the first, the sample model matched the population model (i.e., the covariate was associated with both the p and the three specific factors). In contrast, in the second, I constrained the covariate to only be associated with the general factor (i.e., I fixed the associations between the covariate and the specific factors at zero).

The second column (labeled “Sample model: Covariate -> p & S1-S3”) shows that the model perfectly recovers the simulated association between the covariate and the general factor when all paths are included (i.e., when freely estimating all associations between the covariate and both the p and three specific factors). The third column (labeled “Sample model: Covariate -> p”) shows that the modeled association between the covariate and the general factor was overestimated when fixing the associations between the covariate and the three specific factors at zero. The last column (labeled “Bias when constraining the associations between the covariate and specific factors at zero”) computes the difference, that is, the magnitude of the overestimation, which equaled, on the average, 0.07 standardized units.

| **Table A. Association between a covariate and the** general **factor, as a function of simulated parameters and sample models.** | | | |
| --- | --- | --- | --- |
|  | Mean association between covariate and general factor | |  |
| Simulated population structural model | Sample model: Covariate -> p & S1-S3 | Sample model: Covariate -> p | Bias when constraining the associations between the covariate and specific factors at zero |
| Covariate -> p: 0.80  Covariate -> S1-S3: 0.80 | 0.80 | 0.88 | +0.08 |
| Covariate -> p: 0.80  Covariate -> S1-S3: 0.60 | 0.80 | 0.87 | +0.07 |
| Covariate -> p: 0.80  Covariate -> S1-S3: 0.40 | 0.80 | 0.86 | +0.06 |

To examine this empirically, I re-analyzed a sample of Swedish adults aged 35-45 (N = 909,699) described elsewhere.^2^ Diagnoses on fourteen disorders were retrieved from electronic health records, and coded on a lifetime basis. For simplicity, I selected 9 diagnoses roughly covering internalizing, externalizing, and psychotic problems. I conducted a bifactor confirmatory factor analysis (BCFA) of the 9 diagnoses (Table B below), treating the outcomes as categorical (i.e., I fit the model to the tetrachoric correlation matrix).

I subsequently constrained the factor loadings to that derived from BCFA without a covariate, and then estimated correlations between the latent factors and the sum score based on the 9 diagnoses in two different ways. In the first model, following Fried and colleagues,^1^ I constrained the correlation between the specific factors and the total sum at zero (i.e., I only allowed the total sum score to correlate with the general factor). In this first model, the correlation between the sum score and the general factor equaled *r* = 0.74. In the second model, I allowed the total sum score to correlate with both the general factor and the three specific factors (i.e., the specific internalizing, externalizing, and psychotic factors). Then the correlations between the sum score and the general factor equaled *r* = 0.57. This empirical demonstration highlights that an association between a covariate and a latent factor is likely over-estimated when constraining the covariate to only be associated with one (out of several) latent factors in a multidimensional model with cross-loadings.

| **Table B. Associations between bifactor model and total sum score** | | | | |
| --- | --- | --- | --- | --- |
| **Diagnosis** | **General factor** | **Internalizing factor** | **Externalizing factor** | **Psychotic factor** |
| **Depression** | 0.84 | 0.31 | 0 | 0 |
| **Anxiety** | 0.78 | 0.24 | 0 | 0 |
| **Post-traumatic stress disorder** | 0.66 | 0.40 | 0 | 0 |
| **Drug misuse** | 0.63 | 0 | 0.34 | 0 |
| **Alcohol abuse** | 0.75 | 0 | 0.67 | 0 |
| **Attention-deficit/Hyper-activity disorders** | 0.72 | 0 | 0.15 | 0 |
| **Bipolar** | 0.75 | 0 | 0 | 0.28 |
| **Schizophrenia** | 0.52 | 0 | 0 | 0.86 |
| **Schizoaffective** | 0.46 | 0 | 0 | 0.62 |
| **Correlation between diagnostic sum and latent general factor (when constraining the correlations with the specific factors at zero)** | 0.74 |  |  |  |
| **Correlation between diagnostic sum and latent general factor (when allowing the sum score to correlate freely with all factors)** | 0.57 |  |  |  |

**Supplement 2. On the effect of range restriction on the factor congruence and correlation coefficients.**

Whereas the congruence coefficient can overestimate factor similarity when loadings share the same sign, the correlation coefficient can underestimate similarity when there is range restriction. To illustrate, I simulated two factors with 20 indicators and loadings ranging from 0 to 1, and where the congruence coefficient equaled K=0.98. Although not part of the simulation, the correlation coefficient was also high (*r*=0.89). Figure 1 below displays each indicator as a dot.

When restricting the loading range to that of the general factors based on the bifactor confirmatory factor analyses of the epidemiological samples (such that that the loadings were not less than 0.32 or greater than 0.79, corresponding the indicators in black in Figure 1 above) as reported by Levin-Aspenson and colleagues,^3^ the congruence coefficient remained similar (K=0.97). However, the correlation attenuated (*r*=0.27). To examine this effect in the population, I re-ran the above simulation 1,000 times. Whereas the congruence coefficient remained similar (mean K=0.98), the correlation attenuated to roughly half (mean *r*=0.51).

In conclusion, this simulation implies that for general factors, where loadings typically do not span the entire range, the correlation coefficient might be lower than the congruence coefficient.

**Supplement 3. R-code for rotating a factor analytic solution toward principal components**

eigen.rotation <- function(x){

#REQUIRE PACKAGES

require(psych);require(GPArotation)

#FIRST ROTATE SOLUTION TO VARIMAX TO GET RID OF CORRELATED FACTORS

x <- varimax(x)$loadings

#COMMUNALITY

x.communality <- apply(x^2,1,sum)

#SCALE BY COMMUNALITY

x.scaled <- x/sqrt(x.communality)

#REPRODUCED CORRELATION MATRIX

x.scaled.reproduced.cor <- x.scaled %*% t(x.scaled)

#(STANDARDIZED) EIGEN DECOMPOSITION OF MATRIX

x.eigen <- eigen(x.scaled.reproduced.cor)$vectors[,1:dim(x)[2]] %*% diag(sqrt(eigen(x.scaled.reproduced.cor)$values[1:dim(x)[2]]))

#SCALE BACK BY COMMUNALITY

x.eigen.scaled <- x.eigen * sqrt(x.communality)

#APPLY NAMES TO DATA

rownames(x.eigen.scaled) <- rownames(x)

#RETURN DATA

return(x.eigen.scaled)

}

**Supplementary Table 1A. Confirmatory bifactor factor analyses of 14 psychiatric diagnoses in Swedish population sample (age 35-45).**

|  | **Freely estimated model** | | | | |  | **Fixed and inverted general factor loadings** | | | | |
| --- | --- | --- | --- | --- | --- | --- | --- | --- | --- | --- | --- |
| **Diagnosis** | **General factor** | **Int** | **Ext** | **Neuro** | **Psy** |  | **General factor** | **Int** | **Ext** | **Neuro** | **Psy** |
| **Depression** | 0.79 | 0.47 | 0 | 0 | 0 |  | 0.47 | 0.85 | 0 | 0 | 0 |
| **Anxiety** | 0.77 | 0.34 | 0 | 0 | 0 |  | 0.48 | 0.67 | 0 | 0 | 0 |
| **OCD** | 0.63 | 0.21 | 0 | 0 | 0 |  | 0.62 | 0.32 | 0 | 0 | 0 |
| **PTSD** | 0.62 | 0.34 | 0 | 0 | 0 |  | 0.63 | 0.40 | 0 | 0 | 0 |
| **Eating disorder** | 0.47 | 0.38 | 0 | 0 | 0 |  | 0.79 | 0.09 | 0 | 0 | 0 |
| **Alcohol** | 0.59 | 0 | 0.44 | 0 | 0 |  | 0.65 | 0 | 0.39 | 0 | 0 |
| **Drug** | 0.71 | 0 | 0.63 | 0 | 0 |  | 0.55 | 0 | 0.84 | 0 | 0 |
| **ADHD** | 0.73 | 0 | 0.19 | 0.39 | 0 |  | 0.51 | 0 | 0.44 | 0.59 | 0 |
| **ODD** | 0.48 | 0 | 0.19 | 0.18 | 0 |  | 0.77 | 0 | 0.04 | -0.10 | 0 |
| **Autism** | 0.65 | 0 | 0 | 0.48 | 0 |  | 0.59 | 0 | 0 | 0.59 | 0 |
| **Tics** | 0.51 | 0 | 0 | 0.51 | 0 |  | 0.73 | 0 | 0 | 0.32 | 0 |
| **Bipolar** | 0.68 | 0.24 | 0 | 0 | 0.29 |  | 0.56 | 0.45 | 0 | 0 | 0.36 |
| **Schizoaffective** | 0.56 | 0 | 0 | 0 | 0.83 |  | 0.68 | 0 | 0 | 0 | 0.74 |
| **Schizophrenia** | 0.55 | 0 | 0 | 0 | 0.55 |  | 0.71 | 0 | 0 | 0 | 0.36 |
| *Note.* In the model labeled “Freely estimated model”, all parameters (except those fixed at zero) were estimated. In the model labeled “Fixed and inverted general factor loadings”, the loadings on the general factor were inverted from the “Freely estimated model”.  OCD = Obsessive-compulsive disorder. PTSD = Post-traumatic stress disorder. ADHD = Attention-deficit/hyper-activity disorder. ODD = Oppositional defiant disorder.  The sample is the same as described in Supplement 1.^2^ | | | | | | | | | | | |

**Supplementary Table 1B. 1-factor models of 14 psychiatric diagnoses in Swedish population sample (age 35-45).**

| **Diagnosis** | **Freely estimated general factor** | **Fixed and inverted general factor loadings** |
| --- | --- | --- |
| **Depression** | 0.83 | 0.51 |
| **Anxiety** | 0.79 | 0.52 |
| **OCD** | 0.65 | 0.66 |
| **PTSD** | 0.66 | 0.65 |
| **Eating disorder** | 0.52 | 0.79 |
| **Alcohol** | 0.61 | 0.68 |
| **Drug** | 0.73 | 0.61 |
| **ADHD** | 0.76 | 0.55 |
| **ODD** | 0.51 | 0.83 |
| **Autism** | 0.68 | 0.61 |
| **Tics** | 0.55 | 0.76 |
| **Bipolar** | 0.74 | 0.59 |
| **Schizoaffective** | 0.61 | 0.73 |
| **Schizophrenia** | 0.59 | 0.74 |
| *Note.* In the model labeled “Freely estimated model”, all parameters. In the model labeled “Fixed and inverted general factor loadings”, the loadings on the general factor were inverted from the “Freely estimated general factor”.  OCD = Obsessive-compulsive disorder. PTSD = Post-traumatic stress disorder. ADHD = Attention-deficit/hyper-activity disorder. ODD = Oppositional defiant disorder.  The sample is the same as described in Supplement 1.^2^ | | |

**Supplementary Table 2A. Confirmatory bifactor models of psychiatric conditions, prescribed anti-depressants, and crimes in Swedish male birth cohort 1 (age 51-62).**

|  | **Freely estimated model** | | | |  | **Fixed and inverted general factor loadings** | | | |
| --- | --- | --- | --- | --- | --- | --- | --- | --- | --- |
| **Condition** | **General factor** | **Int** | **Psy** | **Ext** |  | **General factor** | **Int** | **Psy** | **Ext** |
| **Depression** | 0.83 | 0.33 | 0 | 0 |  | 0.29 | 0.96 | 0 | 0 |
| **Anxiety** | 0.75 | 0.36 | 0 | 0 |  | 0.39 | 0.70 | 0 | 0 |
| **OCD** | 0.68 | 0.37 | 0 | 0 |  | 0.53 | 0.30 | 0 | 0 |
| **PTSD** | 0.64 | 0.23 | 0 | 0 |  | 0.64 | 0.4 | 0 | 0 |
| **Anti-depressant prescription** | 0.53 | 0.57 | 0 | 0 |  | 0.68 | 0.62 | 0 | 0 |
| **Bipolar** | 0.49 | 0 | 0.39 | 0 |  | 0.71 | 0 | 0.53 | 0 |
| **Schizophrenia** | 0.54 | 0 | 0.64 | 0 |  | 0.67 | 0 | 0.73 | 0 |
| **Schizoaffective** | 0.63 | 0 | 0.85 | 0 |  | 0.66 | 0 | 0.19 | 0 |
| **Alcohol** | 0.71 | 0 | 0 | 0.43 |  | 0.49 | 0 | 0 | 0.72 |
| **Drugs** | 0.39 | 0 | 0 | 0.47 |  | 0.75 | 0 | 0 | 0.44 |
| **Violent crimes** | 0.29 | 0 | 0 | 0.59 |  | 0.83 | 0 | 0 | 0.07 |
| **Property crimes** | 0.67 | 0 | 0 | 0.68 |  | 0.54 | 0 | 0 | -0.09 |
| **Suicide** | 0.66 | -0.16 | 0 | 0.13 |  | 0.63 | 0.20 | 0 | 0.27 |
| *Note.* In the model labeled “Freely estimated model”, all parameters (except those fixed at zero) were estimated. In the model labeled “Fixed and inverted general factor loadings”, the loadings on the general factor were inverted from the “Freely estimated model”.  Int = Specific internalizing factor. Psy = Specific psychotic factors. Ext = Specific externalizing factor.  OCD = Obsessive-compulsive disorder. PTSD = Post-traumatic stress disorder.  The sample is described elsewhere.^4^ | | | | | | | | | |

**Supplementary Table 2B. 1-factor models of psychiatric conditions, prescribed anti-depressants, and crimes in Swedish male birth cohort 1 (age 51-62).**

| **Condition** | **Freely estimated general factor** | **Fixed and inverted general factor** |
| --- | --- | --- |
| **Depression** | 0.84 | 0.40 |
| **Anxiety** | 0.77 | 0.48 |
| **OCD** | 0.58 | 0.72 |
| **PTSD** | 0.63 | 0.63 |
| **Anti-depressant prescription** | 0.72 | 0.58 |
| **Bipolar** | 0.73 | 0.57 |
| **Schizophrenia** | 0.57 | 0.73 |
| **Schizoaffective** | 0.61 | 0.69 |
| **Alcohol** | 0.72 | 0.60 |
| **Drugs** | 0.69 | 0.61 |
| **Violent crimes** | 0.48 | 0.77 |
| **Property crimes** | 0.40 | 0.84 |
| **Suicide** | 0.60 | 0.72 |
| *Note.* In the model labeled “Freely estimated general factor”, all parameters were estimated. In the model labeled “Fixed and inverted general factor”, the loadings on the general factor were inverted from the “Freely estimated general model”.  Int = Specific internalizing factor. Psy = Specific psychotic factors. Ext = Specific externalizing factor.  OCD = Obsessive-compulsive disorder. PTSD = Post-traumatic stress disorder.  The sample is described elsewhere.^4^ | | |

**Supplementary Table 3A. Confirmatory bifactor models of psychiatric conditions, prescribed anti-depressants, and crimes in Swedish male birth cohort 2 (age 42-62).**

|  | **Freely estimated model** | | | |  | **Fixed and inverted general factor loadings** | | | |
| --- | --- | --- | --- | --- | --- | --- | --- | --- | --- |
| **Condition** | **General factor** | **Int** | **Psy** | **Ext** |  | **General factor** | **Int** | **Psy** | **Ext** |
| **Depression** | 0.84 | 0.31 | 0 | 0 |  | 0.28 | 0.96 | 0 | 0 |
| **Anxiety** | 0.75 | 0.35 | 0 | 0 |  | 0.39 | 0.71 | 0 | 0 |
| **OCD** | 0.57 | 0.39 | 0 | 0 |  | 0.72 | 0.34 | 0 | 0 |
| **PTSD** | 0.67 | 0.19 | 0 | 0 |  | 0.67 | 0.43 | 0 | 0 |
| **Anti-depressant prescription** | 0.72 | 0.58 | 0 | 0 |  | 0.50 | 0.69 | 0 | 0 |
| **Bipolar** | 0.72 | 0 | 0.35 | 0 |  | 0.54 | 0 | 0.46 | 0 |
| **Schizophrenia** | 0.50 | 0 | 0.61 | 0 |  | 0.72 | 0 | 0.37 | 0 |
| **Schizoaffective** | 0.54 | 0 | 0.84 | 0 |  | 0.72 | 0 | 0.69 | 0 |
| **Alcohol** | 0.69 | 0 | 0 | 0.37 |  | 0.66 | 0 | 0 | 0.50 |
| **Drugs** | 0.72 | 0 | 0 | 0.50 |  | 0.57 | 0 | 0 | 0.45 |
| **Violent crimes** | 0.39 | 0 | 0 | 0.55 |  | 0.75 | 0 | 0 | -0.02 |
| **Property crimes** | 0.28 | 0 | 0 | 0.66 |  | 0.84 | 0 | 0 | -0.29 |
| **Suicide** | 0.66 | -0.160 | 0 | 0.16 |  | 0.69 | 0.20 | 0 | 0.43 |
| *Note.* In the model labeled “Freely estimated model”, all parameters (except those fixed at zero) were estimated. In the model labeled “Fixed and inverted general factor loadings”, the loadings on the general factor were inverted from the “Freely estimated model”.  Int = Specific internalizing factor. Psy = Specific psychotic factors. Ext = Specific externalizing factor.  OCD = Obsessive-compulsive disorder. PTSD = Post-traumatic stress disorder.  The sample is described elsewhere.^4^ | | | | | | | | | |

**Supplementary Table 3B. 1-factor models of psychiatric conditions, prescribed anti-depressants, and crimes in Swedish male birth cohort 2 (age 42-62).**

| **Condition** | **Freely estimated general factor** | **Fixed and inverted general factor** |
| --- | --- | --- |
| **Depression** | 0.85 | 0.39 |
| **Anxiety** | 0.78 | 0.57 |
| **OCD** | 0.72 | 0.62 |
| **PTSD** | 0.77 | 0.61 |
| **Anti-depressant prescription** | 0.61 | 0.77 |
| **Bipolar** | 0.57 | 0.78 |
| **Schizophrenia** | 0.62 | 0.72 |
| **Schizoaffective** | 0.67 | 0.67 |
| **Alcohol** | 0.74 | 0.61 |
| **Drugs** | 0.47 | 0.78 |
| **Violent crimes** | 0.39 | 0.85 |
| **Property crimes** | 0.78 | 0.47 |
| **Suicide** | 0.61 | 0.74 |
| *Note.* In the model labeled “Freely estimated general factor”, all parameters were estimated. In the model labeled “Fixed and inverted general factor”, the loadings on the general factor were inverted from the “Freely estimated general model”.  Int = Specific internalizing factor. Psy = Specific psychotic factors. Ext = Specific externalizing factor.  OCD = Obsessive-compulsive disorder. PTSD = Post-traumatic stress disorder.  The sample is described elsewhere.^4^ | | |

**Supplementary Table 4A. Confirmatory bifactor models of psychiatric conditions, prescribed anti-depressants, and crimes in Swedish male birth cohort 3 (age 33-52).**

|  | **Freely estimated model** | | | |  | **Fixed and inverted general factor loadings** | | | |
| --- | --- | --- | --- | --- | --- | --- | --- | --- | --- |
| **Condition** | **General factor** | **Int** | **Psy** | **Ext** |  | **General factor** | **Int** | **Psy** | **Ext** |
| **Depression** | 0.86 | 0.24 | 0 | 0 |  | 0.31 | 0.95 | 0 | 0 |
| **Anxiety** | 0.78 | 0.33 | 0 | 0 |  | 0.55 | 0.62 | 0 | 0 |
| **OCD** | 0.58 | 0.47 | 0 | 0 |  | 0.74 | 0.36 | 0 | 0 |
| **PTSD** | 0.70 | 0.07 | 0 | 0 |  | 0.63 | 0.42 | 0 | 0 |
| **Anti-depressant prescription** | 0.79 | 0.51 | 0 | 0 |  | 0.39 | 0.81 | 0 | 0 |
| **Bipolar** | 0.74 | 0 | 0.20 | 0 |  | 0.58 | 0 | 0.29 | 0 |
| **Schizophrenia** | 0.55 | 0 | 0.55 | 0 |  | 0.78 | 0 | 0.26 | 0 |
| **Schizoaffective** | 0.59 | 0 | 0.81 | 0 |  | 0.73 | 0 | 0.68 | 0 |
| **Alcohol** | 0.63 | 0 | 0 | 0.36 |  | 0.70 | 0 | 0 | 0.44 |
| **Drugs** | 0.73 | 0 | 0 | 0.52 |  | 0.59 | 0 | 0 | 0.38 |
| **Violent crimes** | 0.39 | 0 | 0 | 0.56 |  | 0.79 | 0 | 0 | -0.07 |
| **Property crimes** | 0.31 | 0 | 0 | 0.65 |  | 0.86 | 0 | 0 | -0.33 |
| **Suicide** | 0.64 | -0.28 | 0 | 0.20 |  | 0.64 | 0.15 | 0 | 0.49 |
| *Note.* In the model labeled “Freely estimated model”, all parameters (except those fixed at zero) were estimated. In the model labeled “Fixed and inverted general factor loadings”, the loadings on the general factor were inverted from the “Freely estimated model”.  Int = Specific internalizing factor. Psy = Specific psychotic factors. Ext = Specific externalizing factor.  OCD = Obsessive-compulsive disorder. PTSD = Post-traumatic stress disorder.  The sample is described elsewhere.^4^ | | | | | | | | | |

**Supplementary Table 4B. 1-factor models of psychiatric conditions, prescribed anti-depressants, and crimes in Swedish male birth cohort 3 (age 33-52).**

| **Condition** | **Freely estimated general factor** | **Fixed and inverted general factor** |
| --- | --- | --- |
| **Depression** | 0.86 | 0.41 |
| **Anxiety** | 0.80 | 0.59 |
| **OCD** | 0.62 | 0.74 |
| **PTSD** | 0.68 | 0.64 |
| **Anti-depressant prescription** | 0.82 | 0.48 |
| **Bipolar** | 0.74 | 0.62 |
| **Schizophrenia** | 0.60 | 0.79 |
| **Schizoaffective** | 0.64 | 0.68 |
| **Alcohol** | 0.67 | 0.67 |
| **Drugs** | 0.79 | 0.60 |
| **Violent crimes** | 0.48 | 0.82 |
| **Property crimes** | 0.41 | 0.86 |
| **Suicide** | 0.59 | 0.80 |
| *Note.* In the model labeled “Freely estimated general factor”, all parameters were estimated. In the model labeled “Fixed and inverted general factor”, the loadings on the general factor were inverted from the “Freely estimated general model”.  Int = Specific internalizing factor. Psy = Specific psychotic factors. Ext = Specific externalizing factor.  OCD = Obsessive-compulsive disorder. PTSD = Post-traumatic stress disorder.  The sample is described elsewhere.^4^ | | |

**Supplementary Table 5A. Confirmatory bifactor models of psychiatric conditions, prescribed anti-depressants, and crimes in Swedish male birth cohort 4 (age 23-40).**

|  | **Freely estimated model** | | | |  | **Fixed and inverted general factor loadings** | | | |
| --- | --- | --- | --- | --- | --- | --- | --- | --- | --- |
| **Condition** | **General factor** | **Int** | **Psy** | **Ext** |  | **General factor** | **Int** | **Psy** | **Ext** |
| **Depression** | 0.89 | 0.17 | 0 | 0 |  | 0.35 | 0.86 | 0 | 0 |
| **Anxiety** | 0.80 | 0.32 | 0 | 0 |  | 0.51 | 0.66 | 0 | 0 |
| **OCD** | 0.51 | 0.67 | 0 | 0 |  | 0.80 | 0.32 | 0 | 0 |
| **PTSD** | 0.71 | -0.02 | 0 | 0 |  | 0.56 | 0.44 | 0 | 0 |
| **Anti-depressant prescription** | 0.87 | 0.40 | 0 | 0 |  | 0.35 | 0.93 | 0 | 0 |
| **Bipolar** | 0.75 | 0 | 0.22 | 0 |  | 0.51 | 0 | 0.42 | 0 |
| **Schizophrenia** | 0.56 | 0 | 0.48 | 0 |  | 0.71 | 0 | 0.35 | 0 |
| **Schizoaffective** | 0.60 | 0 | 0.80 | 0 |  | 0.60 | 0 | 0.80 | 0 |
| **Alcohol** | 0.51 | 0 | 0 | 0.39 |  | 0.75 | 0 | 0 | 0.66 |
| **Drugs** | 0.71 | 0 | 0 | 0.52 |  | 0.54 | 0 | 0 | 0.22 |
| **Violent crimes** | 0.35 | 0 | 0 | 0.58 |  | 0.87 | 0 | 0 | -0.23 |
| **Property crimes** | 0.35 | 0 | 0 | 0.62 |  | 0.89 | 0 | 0 | -0.35 |
| **Suicide** | 0.54 | -0.15 | 0 | 0.28 |  | 0.71 | 0.09 | 0 | 0.25 |
| *Note.* In the model labeled “Freely estimated model”, all parameters (except those fixed at zero) were estimated. In the model labeled “Fixed and inverted general factor loadings”, the loadings on the general factor were inverted from the “Freely estimated model”.  Int = Specific internalizing factor. Psy = Specific psychotic factors. Ext = Specific externalizing factor.  OCD = Obsessive-compulsive disorder. PTSD = Post-traumatic stress disorder.  The sample is described elsewhere.^4^ | | | | | | | | | |

**Supplementary Table 5B. 1-factor models of psychiatric conditions, prescribed anti-depressants, and crimes in Swedish male birth cohort 4 (age 23-40).**

| **Condition** | **Freely estimated general factor** | **Fixed and inverted general factor** |
| --- | --- | --- |
| **Depression** | 0.87 | 0.45 |
| **Anxiety** | 0.81 | 0.55 |
| **OCD** | 0.57 | 0.78 |
| **PTSD** | 0.67 | 0.60 |
| **Anti-depressant prescription** | 0.88 | 0.44 |
| **Bipolar** | 0.75 | 0.57 |
| **Schizophrenia** | 0.60 | 0.67 |
| **Schizoaffective** | 0.65 | 0.65 |
| **Alcohol** | 0.57 | 0.75 |
| **Drugs** | 0.78 | 0.57 |
| **Violent crimes** | 0.44 | 0.88 |
| **Property crimes** | 0.45 | 0.87 |
| **Suicide** | 0.55 | 0.81 |
| *Note.* In the model labeled “Freely estimated general factor”, all parameters were estimated. In the model labeled “Fixed and inverted general factor”, the loadings on the general factor were inverted from the “Freely estimated general model”.  Int = Specific internalizing factor. Psy = Specific psychotic factors. Ext = Specific externalizing factor.  OCD = Obsessive-compulsive disorder. PTSD = Post-traumatic stress disorder.  The sample is described elsewhere.^4^ | | |

**Supplementary Table 6A. Confirmatory bifactor factor analysis of 62 symptoms in the CATSS sample (age 9).**

|  | **Factors** | | | | | | | |
| --- | --- | --- | --- | --- | --- | --- | --- | --- |
| **Item** | **General factor** | **Inattention** | **Impulsivity** | **Learning** | **Autism** | **Tics** | **Conduct** | **Anxiety** |
| Does s/he often fail to pay close attention to details or make careless mistakes in schoolwork, or other activities? | 0.69 | 0.45 | 0 | 0 | 0 | 0 | 0 | 0 |
| Does s/he often have difficulty sustaining attention in tasks or play activities? | 0.79 | 0.43 | 0 | 0 | 0 | 0 | 0 | 0 |
| Does s/he often seem not to listen when spoken to directly? | 0.78 | 0.28 | 0 | 0 | 0 | 0 | 0 | 0 |
| Does s/he have difficulty following instructions and to finish tasks? | 0.83 | 0.33 | 0 | 0 | 0 | 0 | 0 | 0 |
| Does s/he often have difficulty organizing tasks and activities? | 0.83 | 0.32 | 0 | 0 | 0 | 0 | 0 | 0 |
| Does s/he often avoid tasks that require sustained mental effort (such as homework)? | 0.74 | 0.31 | 0 | 0 | 0 | 0 | 0 | 0 |
| Does s/he often lose things? | 0.72 | 0.39 | 0 | 0 | 0 | 0 | 0 | 0 |
| Is s/he often easily distracted or disturbed? | 0.81 | 0.29 | 0 | 0 | 0 | 0 | 0 | 0 |
| Is s/he often forgetful in daily activities? | 0.68 | 0.50 | 0 | 0 | 0 | 0 | 0 | 0 |
| Does s/he have difficulties holding his/her hands and feet still or can s/he not stay seated? | 0.63 | 0 | 0.50 | 0 | 0 | 0 | 0 | 0 |
| Does s/he get up and move about in school or in other situations when s/he is supposed to remain seated? | 0.70 | 0 | 0.38 | 0 | 0 | 0 | 0 | 0 |
| Does s/he often run around and climbs more than his/hers peers? | 0.59 | 0 | 0.54 | 0 | 0 | 0 | 0 | 0 |
| Does s/he have difficulty playing calmly and quietly? | 0.73 | 0 | 0.48 | 0 | 0 | 0 | 0 | 0 |
| Is s/he often”on the go” or does s/he often act as if ”driven by a motor”? | 0.65 | 0 | 0.61 | 0 | 0 | 0 | 0 | 0 |
| Does s/he talk excessively? | 0.51 | 0 | 0.55 | 0 | 0 | 0 | 0 | 0 |
| Does s/he often blurt out answers before the question has been completed? | 0.56 | 0 | 0.55 | 0 | 0 | 0 | 0 | 0 |
| Does s/he have difficulty awaiting turns? | 0.70 | 0 | 0.53 | 0 | 0 | 0 | 0 | 0 |
| Does s/he often interrupt or intrude on others? | 0.69 | 0 | 0.52 | 0 | 0 | 0 | 0 | 0 |
| Does s/he easily get bored? | 0.67 | 0 | 0.28 | 0 | 0 | 0 | 0 | 0 |
| Has s/he had more difficulties than expected acquiring reading skills? | 0.54 | 0 | 0 | 0.61 | 0 | 0 | 0 | 0 |
| Is learning slow and laborious? | 0.72 | 0 | 0 | 0.56 | 0 | 0 | 0 | 0 |
| Does s/he have difficulties with basic maths? | 0.58 | 0 | 0 | 0.59 | 0 | 0 | 0 | 0 |
| Does s/he have difficulty shifting plan or strategy when this is required? | 0.79 | 0 | 0 | -0.06 | 0 | 0 | 0 | 0 |
| Does s/he have difficulty keeping basic order around him/her? | 0.74 | 0 | 0 | -0.26 | 0 | 0 | 0 | 0 |
| Does s/he have difficulties remembering where s/he put things? | 0.77 | 0 | 0 | -0.24 | 0 | 0 | 0 | 0 |
| Does s/he have difficulties remembering long or multiple-step instructions? | 0.82 | 0 | 0 | 0.26 | 0 | 0 | 0 | 0 |
| Does s/he have difficulties learning rhymes, songs, multiplication tables etc by heart? | 0.62 | 0 | 0 | 0.59 | 0 | 0 | 0 | 0 |
| Was his/her language development delayed or does s/he not speak at all? If one does not start speaking around age 4-5 one is late. Big problems speaking clearly count. | 0.43 | 0 | 0 | 0 | 0.30 | 0 | 0 | 0 |
| Does s/he have difficulties sustaining a conversation? | 0.68 | 0 | 0 | 0 | 0.47 | 0 | 0 | 0 |
| Does s/he like to repeat words and expressions or does s/he use words in a way other people find strange? | 0.63 | 0 | 0 | 0 | 0.39 | 0 | 0 | 0 |
| Has s/he difficulties with pretend play or does s/he imitate considerably less than other children? | 0.57 | 0 | 0 | 0 | 0.43 | 0 | 0 | 0 |
| Does s/he talk in too high a pitch or too quietly? | 0.45 | 0 | 0 | 0 | 0.22 | 0 | 0 | 0 |
| Does s/he have difficulties keeping "on track" when telling other people something? | 0.71 | 0 | 0 | 0 | 0.16 | 0 | 0 | 0 |
| Does s/he have difficulties expressing emotions and reactions with facial gestures, prosody, or body language? | 0.67 | 0 | 0 | 0 | 0.56 | 0 | 0 | 0 |
| Does s/he exhibit considerable difficulties interacting with peers? | 0.70 | 0 | 0 | 0 | 0.55 | 0 | 0 | 0 |
| Is s/he uninterested in sharing joy, interests, and activities with others? | 0.68 | 0 | 0 | 0 | 0.54 | 0 | 0 | 0 |
| Can s/he only be with other people on his/her terms? | 0.67 | 0 | 0 | 0 | 0.42 | 0 | 0 | 0 |
| Does s/he have difficulties behaving as expected by peers? | 0.79 | 0 | 0 | 0 | 0.42 | 0 | 0 | 0 |
| Do other people easily influence him/her? | 0.69 | 0 | 0 | 0 | -0.13 | 0 | 0 | 0 |
| Does s/he get absorbed by his/her interests in such a way as being repetitive or too intense? | 0.65 | 0 | 0 | 0 | 0.32 | 0 | 0 | 0 |
| Does s/he get absorbed by routines in such a way as to produce problems for himself or for other? | 0.67 | 0 | 0 | 0 | 0.44 | 0 | 0 | 0 |
| Has s/he ever engaged in strange hand movements or walking high on tiptoe when s/he was happy or upset? | 0.51 | 0 | 0 | 0 | 0.37 | 0 | 0 | 0 |
| Does s/he get absorbed by details? | 0.68 | 0 | 0 | 0 | 0.32 | 0 | 0 | 0 |
| Does s/he dislike changes in daily routines? | 0.64 | 0 | 0 | 0 | 0.32 | 0 | 0 | 0 |
| Does s/he make unmotivated sounds such as throat clearing, sneezing, swallowing, barking, or shouting? | 0.41 | 0 | 0 | 0 | 0 | 0.91 | 0 | 0 |
| Does s/he make involuntary facial grimaces or body movements? | 0.37 | 0 | 0 | 0 | 0 | 0.57 | 0 | 0 |
| Does s/he make a lot of noise, e.g. whistle, hum, mumble? | 0.60 | 0 | 0 | 0 | 0 | 0.29 | 0 | 0 |
| Has there ever been a time when s/he would be angry to the extent that s/he cannot be reached? | 0.58 | 0 | 0 | 0 | 0 | 0 | 0.37 | 0 |
| Does s/he often argue with adults? | 0.70 | 0 | 0 | 0 | 0 | 0 | 0.42 | 0 |
| Does s/he often tease others by deliberately doing things that are perceived as provocative? | 0.65 | 0 | 0 | 0 | 0 | 0 | 0.40 | 0 |
| Is s/he easily offended, or disturbed by others? | 0.68 | 0 | 0 | 0 | 0 | 0 | 0.44 | 0 |
| Is s/he easily teased? | 0.67 | 0 | 0 | 0 | 0 | 0 | 0.54 | 0 |
| Has s/he ever deliberately been physically cruel to anybody? | 0.58 | 0 | 0 | 0 | 0 | 0 | 0.54 | 0 |
| Does s/he often start fights? | 0.63 | 0 | 0 | 0 | 0 | 0 | 0.55 | 0 |
| Does s/he often lie or cheat? | 0.63 | 0 | 0 | 0 | 0 | 0 | 0.25 | 0 |
| Does s/he steal things at home or outside home? | 0.68 | 0 | 0 | 0 | 0 | 0 | 0.35 | 0 |
| Has s/he ever engaged in shoplifting? | 0.35 | 0 | 0 | 0 | 0 | 0 | 0.30 | 0 |
| Does s/he have panic attacks with sudden strong fear or anxiety? | 0.45 | 0 | 0 | 0 | 0 | 0 | 0 | 0.64 |
| Does s/he fear leaving the house alone, being in crowds, waiting in line or going on a bus or train? | 0.48 | 0 | 0 | 0 | 0 | 0 | 0 | 0.41 |
| Is s/he often particularly nervous or anxious? | 0.56 | 0 | 0 | 0 | 0 | 0 | 0 | 0.59 |
| Does s/he have obsessive thoughts, i.e. thoughts that recur over and over again and that s/he can not stop, for example about dirt, contagion or that something terrible will happen? | 0.48 | 0 | 0 | 0 | 0 | 0 | 0 | 0.73 |
| Does s/he have compulsive behaviours such as washing hands, touch things, control things, repeat things or procedures, arrange or ordering thing, or counting? | 0.50 | 0 | 0 | 0 | 0 | 0 | 0 | 0.61 |
| *Note.* The specific factors were assigned according to the instrument scales.  CATSS = Child and Adolescent Twin Study in Sweden.^5^ | | | | | | | | |

**Supplementary Table 6B. Confirmatory bifactor factor analysis of 62 symptoms in the CATSS sample (age 9), after fixing the loadings on the general factor in an inverted order.**

|  | **Factors** | | | | | | | |
| --- | --- | --- | --- | --- | --- | --- | --- | --- |
| **Item** | **General factor** | **Inattention** | **Impulsivity** | **Learning** | **Autism** | **Tics** | **Conduct** | **Anxiety** |
| Does s/he often fail to pay close attention to details or make careless mistakes in schoolwork, or other activities? | 0.62 | 0.58 | 0 | 0 | 0 | 0 | 0 | 0 |
| Does s/he often have difficulty sustaining attention in tasks or play activities? | 0.45 | 0.79 | 0 | 0 | 0 | 0 | 0 | 0 |
| Does s/he often seem not to listen when spoken to directly? | 0.48 | 0.67 | 0 | 0 | 0 | 0 | 0 | 0 |
| Does s/he have difficulty following instructions and to finish tasks? | 0.37 | 0.83 | 0 | 0 | 0 | 0 | 0 | 0 |
| Does s/he often have difficulty organizing tasks and activities? | 0.35 | 0.84 | 0 | 0 | 0 | 0 | 0 | 0 |
| Does s/he often avoid tasks that require sustained mental effort (such as homework)? | 0.51 | 0.64 | 0 | 0 | 0 | 0 | 0 | 0 |
| Does s/he often lose things? | 0.56 | 0.55 | 0 | 0 | 0 | 0 | 0 | 0 |
| Is s/he often easily distracted or disturbed? | 0.43 | 0.75 | 0 | 0 | 0 | 0 | 0 | 0 |
| Is s/he often forgetful in daily activities? | 0.63 | 0.55 | 0 | 0 | 0 | 0 | 0 | 0 |
| Does s/he have difficulties holding his/her hands and feet still or can s/he not stay seated? | 0.68 | 0 | 0.44 | 0 | 0 | 0 | 0 | 0 |
| Does s/he get up and move about in school or in other situations when s/he is supposed to remain seated? | 0.58 | 0 | 0.53 | 0 | 0 | 0 | 0 | 0 |
| Does s/he often run around and climbs more than his/hers peers? | 0.70 | 0 | 0.42 | 0 | 0 | 0 | 0 | 0 |
| Does s/he have difficulty playing calmly and quietly? | 0.54 | 0 | 0.71 | 0 | 0 | 0 | 0 | 0 |
| Is s/he often”on the go” or does s/he often act as if ”driven by a motor”? | 0.67 | 0 | 0.58 | 0 | 0 | 0 | 0 | 0 |
| Does s/he talk excessively? | 0.74 | 0 | 0.25 | 0 | 0 | 0 | 0 | 0 |
| Does s/he often blurt out answers before the question has been completed? | 0.72 | 0 | 0.35 | 0 | 0 | 0 | 0 | 0 |
| Does s/he have difficulty awaiting turns? | 0.58 | 0 | 0.68 | 0 | 0 | 0 | 0 | 0 |
| Does s/he often interrupt or intrude on others? | 0.63 | 0 | 0.58 | 0 | 0 | 0 | 0 | 0 |
| Does s/he easily get bored? | 0.67 | 0 | 0.29 | 0 | 0 | 0 | 0 | 0 |
| Has s/he had more difficulties than expected acquiring reading skills? | 0.73 | 0 | 0 | 0.33 | 0 | 0 | 0 | 0 |
| Is learning slow and laborious? | 0.56 | 0 | 0 | 0.66 | 0 | 0 | 0 | 0 |
| Does s/he have difficulties with basic maths? | 0.70 | 0 | 0 | 0.39 | 0 | 0 | 0 | 0 |
| Does s/he have difficulty shifting plan or strategy when this is required? | 0.48 | 0 | 0 | 0.38 | 0 | 0 | 0 | 0 |
| Does s/he have difficulty keeping basic order around him/her? | 0.51 | 0 | 0 | 0.49 | 0 | 0 | 0 | 0 |
| Does s/he have difficulties remembering where s/he put things? | 0.50 | 0 | 0 | 0.54 | 0 | 0 | 0 | 0 |
| Does s/he have difficulties remembering long or multiple-step instructions? | 0.41 | 0 | 0 | 0.82 | 0 | 0 | 0 | 0 |
| Does s/he have difficulties learning rhymes, songs, multiplication tables etc by heart? | 0.69 | 0 | 0 | 0.45 | 0 | 0 | 0 | 0 |
| Was his/her language development delayed or does s/he not speak at all? If one does not start speaking around age 4-5 one is late. Big problems speaking clearly count. | 0.81 | 0 | 0 | 0 | -0.17 | 0 | 0 | 0 |
| Does s/he have difficulties sustaining a conversation? | 0.65 | 0 | 0 | 0 | 0.45 | 0 | 0 | 0 |
| Does s/he like to repeat words and expressions or does s/he use words in a way other people find strange? | 0.68 | 0 | 0 | 0 | 0.35 | 0 | 0 | 0 |
| Has s/he difficulties with pretend play or does s/he imitate considerably less than other children? | 0.71 | 0 | 0 | 0 | 0.27 | 0 | 0 | 0 |
| Does s/he talk in too high a pitch or too quietly? | 0.79 | 0 | 0 | 0 | -0.16 | 0 | 0 | 0 |
| Does s/he have difficulties keeping "on track" when telling other people something? | 0.57 | 0 | 0 | 0 | 0.37 | 0 | 0 | 0 |
| Does s/he have difficulties expressing emotions and reactions with facial gestures, prosody, or body language? | 0.65 | 0 | 0 | 0 | 0.54 | 0 | 0 | 0 |
| Does s/he exhibit considerable difficulties interacting with peers? | 0.59 | 0 | 0 | 0 | 0.67 | 0 | 0 | 0 |
| Is s/he uninterested in sharing joy, interests, and activities with others? | 0.65 | 0 | 0 | 0 | 0.56 | 0 | 0 | 0 |
| Can s/he only be with other people on his/her terms? | 0.67 | 0 | 0 | 0 | 0.48 | 0 | 0 | 0 |
| Does s/he have difficulties behaving as expected by peers? | 0.45 | 0 | 0 | 0 | 0.87 | 0 | 0 | 0 |
| Do other people easily influence him/her? | 0.60 | 0 | 0 | 0 | 0.14 | 0 | 0 | 0 |
| Does s/he get absorbed by his/her interests in such a way as being repetitive or too intense? | 0.68 | 0 | 0 | 0 | 0.32 | 0 | 0 | 0 |
| Does s/he get absorbed by routines in such a way as to produce problems for himself or for other? | 0.67 | 0 | 0 | 0 | 0.44 | 0 | 0 | 0 |
| Has s/he ever engaged in strange hand movements or walking high on tiptoe when s/he was happy or upset? | 0.74 | 0 | 0 | 0 | 0.09 | 0 | 0 | 0 |
| Does s/he get absorbed by details? | 0.63 | 0 | 0 | 0 | 0.41 | 0 | 0 | 0 |
| Does s/he dislike changes in daily routines? | 0.68 | 0 | 0 | 0 | 0.33 | 0 | 0 | 0 |
| Does s/he make unmotivated sounds such as throat clearing, sneezing, swallowing, barking, or shouting? | 0.82 | 0 | 0 | 0 | 0 | -0.06 | 0 | 0 |
| Does s/he make involuntary facial grimaces or body movements? | 0.83 | 0 | 0 | 0 | 0 | -0.27 | 0 | 0 |
| Does s/he make a lot of noise, e.g. whistle, hum, mumble? | 0.69 | 0 | 0 | 0 | 0 | 0.70 | 0 | 0 |
| Has there ever been a time when s/he would be angry to the extent that s/he cannot be reached? | 0.70 | 0 | 0 | 0 | 0 | 0 | 0.24 | 0 |
| Does s/he often argue with adults? | 0.58 | 0 | 0 | 0 | 0 | 0 | 0.59 | 0 |
| Does s/he often tease others by deliberately doing things that are perceived as provocative? | 0.68 | 0 | 0 | 0 | 0 | 0 | 0.37 | 0 |
| Is s/he easily offended, or disturbed by others? | 0.63 | 0 | 0 | 0 | 0 | 0 | 0.52 | 0 |
| Is s/he easily teased? | 0.67 | 0 | 0 | 0 | 0 | 0 | 0.55 | 0 |
| Has s/he ever deliberately been physically cruel to anybody? | 0.70 | 0 | 0 | 0 | 0 | 0 | 0.33 | 0 |
| Does s/he often start fights? | 0.69 | 0 | 0 | 0 | 0 | 0 | 0.44 | 0 |
| Does s/he often lie or cheat? | 0.68 | 0 | 0 | 0 | 0 | 0 | 0.15 | 0 |
| Does s/he steal things at home or outside home? | 0.64 | 0 | 0 | 0 | 0 | 0 | 0.34 | 0 |
| Has s/he ever engaged in shoplifting? | 0.83 | 0 | 0 | 0 | 0 | 0 | -0.43 | 0 |
| Does s/he have panic attacks with sudden strong fear or anxiety? | 0.79 | 0 | 0 | 0 | 0 | 0 | 0 | 0.04 |
| Does s/he fear leaving the house alone, being in crowds, waiting in line or going on a bus or train? | 0.79 | 0 | 0 | 0 | 0 | 0 | 0 | -0.57 |
| Is s/he often particularly nervous or anxious? | 0.72 | 0 | 0 | 0 | 0 | 0 | 0 | -0.08 |
| Does s/he have obsessive thoughts, i.e. thoughts that recur over and over again and that s/he can not stop, for example about dirt, contagion or that something terrible will happen? | 0.78 | 0 | 0 | 0 | 0 | 0 | 0 | 0.36 |
| Does s/he have compulsive behaviours such as washing hands, touch things, control things, repeat things or procedures, arrange or ordering thing, or counting? | 0.77 | 0 | 0 | 0 | 0 | 0 | 0 | 0.42 |
| *Note.* The specific factors were assigned according to the instrument scales. | | | | | | | | |

CATSS = Child and Adolescent Twin Study in Sweden.^5^

**Supplementary Table 7C. 1-factor model of 62 symptoms in the CATSS sample (age 9) before and after fixing the loadings on the general factor in an inverted order.**

| **Item** | **General factor** | **Inverted general factor** |
| --- | --- | --- |
| Does s/he often fail to pay close attention to details or make careless mistakes in schoolwork, or other activities? | 0.74 | 0.65 |
| Does s/he often have difficulty sustaining attention in tasks or play activities? | 0.84 | 0.42 |
| Does s/he often seem not to listen when spoken to directly? | 0.80 | 0.47 |
| Does s/he have difficulty following instructions and to finish tasks? | 0.85 | 0.37 |
| Does s/he often have difficulty organizing tasks and activities? | 0.85 | 0.37 |
| Does s/he often avoid tasks that require sustained mental effort (such as homework)? | 0.76 | 0.57 |
| Does s/he often lose things? | 0.76 | 0.58 |
| Is s/he often easily distracted or disturbed? | 0.83 | 0.44 |
| Is s/he often forgetful in daily activities? | 0.74 | 0.64 |
| Does s/he have difficulties holding his/her hands and feet still or can s/he not stay seated? | 0.70 | 0.69 |
| Does s/he get up and move about in school or in other situations when s/he is supposed to remain seated? | 0.74 | 0.63 |
| Does s/he often run around and climbs more than his/hers peers? | 0.68 | 0.70 |
| Does s/he have difficulty playing calmly and quietly? | 0.79 | 0.50 |
| Is s/he often”on the go” or does s/he often act as if ”driven by a motor”? | 0.76 | 0.59 |
| Does s/he talk excessively? | 0.59 | 0.76 |
| Does s/he often blurt out answers before the question has been completed? | 0.64 | 0.74 |
| Does s/he have difficulty awaiting turns? | 0.78 | 0.51 |
| Does s/he often interrupt or intrude on others? | 0.76 | 0.56 |
| Does s/he easily get bored? | 0.69 | 0.70 |
| Has s/he had more difficulties than expected acquiring reading skills? | 0.57 | 0.76 |
| Is learning slow and laborious? | 0.75 | 0.62 |
| Does s/he have difficulties with basic maths? | 0.61 | 0.75 |
| Does s/he have difficulty shifting plan or strategy when this is required? | 0.75 | 0.59 |
| Does s/he have difficulty keeping basic order around him/her? | 0.71 | 0.67 |
| Does s/he have difficulties remembering where s/he put things? | 0.73 | 0.65 |
| Does s/he have difficulties remembering long or multiple-step instructions? | 0.81 | 0.47 |
| Does s/he have difficulties learning rhymes, songs, multiplication tables etc by heart? | 0.65 | 0.74 |
| Was his/her language development delayed or does s/he not speak at all? If one does not start speaking around age 4-5 one is late. Big problems speaking clearly count. | 0.44 | 0.83 |
| Does s/he have difficulties sustaining a conversation? | 0.73 | 0.66 |
| Does s/he like to repeat words and expressions or does s/he use words in a way other people find strange? | 0.66 | 0.71 |
| Has s/he difficulties with pretend play or does s/he imitate considerably less than other children? | 0.61 | 0.75 |
| Does s/he talk in too high a pitch or too quietly? | 0.46 | 0.83 |
| Does s/he have difficulties keeping "on track" when telling other people something? | 0.70 | 0.69 |
| Does s/he have difficulties expressing emotions and reactions with facial gestures, prosody, or body language? | 0.75 | 0.61 |
| Does s/he exhibit considerable difficulties interacting with peers? | 0.77 | 0.54 |
| Is s/he uninterested in sharing joy, interests, and activities with others? | 0.75 | 0.61 |
| Can s/he only be with other people on his/her terms? | 0.71 | 0.66 |
| Does s/he have difficulties behaving as expected by peers? | 0.83 | 0.46 |
| Do other people easily influence him/her? | 0.65 | 0.73 |
| Does s/he get absorbed by his/her interests in such a way as being repetitive or too intense? | 0.66 | 0.71 |
| Does s/he get absorbed by routines in such a way as to produce problems for himself or for other? | 0.71 | 0.66 |
| Has s/he ever engaged in strange hand movements or walking high on tiptoe when s/he was happy or upset? | 0.54 | 0.77 |
| Does s/he get absorbed by details? | 0.70 | 0.69 |
| Does s/he dislike changes in daily routines? | 0.66 | 0.73 |
| Does s/he make unmotivated sounds such as throat clearing, sneezing, swallowing, barking, or shouting? | 0.42 | 0.84 |
| Does s/he make involuntary facial grimaces or body movements? | 0.37 | 0.85 |
| Does s/he make a lot of noise, e.g. whistle, hum, mumble? | 0.58 | 0.76 |
| Has there ever been a time when s/he would be angry to the extent that s/he cannot be reached? | 0.59 | 0.75 |
| Does s/he often argue with adults? | 0.72 | 0.66 |
| Does s/he often tease others by deliberately doing things that are perceived as provocative? | 0.66 | 0.72 |
| Is s/he easily offended, or disturbed by others? | 0.70 | 0.68 |
| Is s/he easily teased? | 0.69 | 0.70 |
| Has s/he ever deliberately been physically cruel to anybody? | 0.62 | 0.75 |
| Does s/he often start fights? | 0.67 | 0.71 |
| Does s/he often lie or cheat? | 0.63 | 0.74 |
| Does s/he steal things at home or outside home? | 0.69 | 0.70 |
| Has s/he ever engaged in shoplifting? | 0.37 | 0.85 |
| Does s/he have panic attacks with sudden strong fear or anxiety? | 0.47 | 0.80 |
| Does s/he fear leaving the house alone, being in crowds, waiting in line or going on a bus or train? | 0.47 | 0.81 |
| Is s/he often particularly nervous or anxious? | 0.56 | 0.76 |
| Does s/he have obsessive thoughts, i.e. thoughts that recur over and over again and that s/he can not stop, for example about dirt, contagion or that something terrible will happen? | 0.50 | 0.79 |
| Does s/he have compulsive behaviours such as washing hands, touch things, control things, repeat things or procedures, arrange or ordering thing, or counting? | 0.51 | 0.78 |

*Note.* CATSS = Child and Adolescent Twin Study in Sweden.^5^

**Supplementary Table 8A. Confirmatory bifactor factor analyses of 74 Adult Behavior Checklist symptoms in the CATSS sample (age 18).**

|  | **Factors** | | | | | | |
| --- | --- | --- | --- | --- | --- | --- | --- |
| **Item** | **General factor** | **Attention Problems** | **Aggressive Behavior** | **Rule-Breaking Behavior** | **Intrusive** | **Anxious/ Depressed** | **Withdrawn** |
| Is too forgetful | 0.43 | 0.71 | 0 | 0 | 0 | 0 | 0 |
| Can’t concentrate, can’t pay attention for long | 0.65 | 0.37 | 0 | 0 | 0 | 0 | 0 |
| Too dependent on others | 0.66 | -0.02 | 0 | 0 | 0 | 0 | 0 |
| Confused or seems to be in a fog | 0.70 | 0.25 | 0 | 0 | 0 | 0 | 0 |
| Daydreams or gets lost in his/her thoughts | 0.48 | 0.24 | 0 | 0 | 0 | 0 | 0 |
| Has trouble planning for the future | 0.69 | 0.24 | 0 | 0 | 0 | 0 | 0 |
| Fails to finish things he/she should do | 0.72 | 0.42 | 0 | 0 | 0 | 0 | 0 |
| Poor work performance | 0.72 | 0.33 | 0 | 0 | 0 | 0 | 0 |
| Has trouble setting priorities | 0.73 | 0.39 | 0 | 0 | 0 | 0 | 0 |
| Has trouble making decisions | 0.68 | 0.16 | 0 | 0 | 0 | 0 | 0 |
| Passive or lacks initiative | 0.72 | 0.13 | 0 | 0 | 0 | 0 | 0 |
| Stays away from school/job even when not sick and not on vacation | 0.62 | 0.19 | 0 | 0 | 0 | 0 | 0 |
| Underactive, slow moving, or lacks energy | 0.70 | 0.11 | 0 | 0 | 0 | 0 | 0 |
| Is disorganized | 0.61 | 0.33 | 0 | 0 | 0 | 0 | 0 |
| Tends to lose things | 0.40 | 0.62 | 0 | 0 | 0 | 0 | 0 |
| He/she is not good at details | 0.56 | 0.40 | 0 | 0 | 0 | 0 | 0 |
| Tends to be late for appointments | 0.49 | 0.42 | 0 | 0 | 0 | 0 | 0 |
| Argues a lot | 0.07 | 0 | 0.50 | 0 | 0 | 0 | 0 |
| Blames others for own problems | 0.56 | 0 | 0.32 | 0 | 0 | 0 | 0 |
| Cruelty, bullying, or meanness to others | 0.59 | 0 | 0.32 | 0 | 0 | 0 | 0 |
| Gets along badly with family | 0.66 | 0 | 0.39 | 0 | 0 | 0 | 0 |
| Gets in many fights | 0.73 | 0 | 0.06 | 0 | 0 | 0 | 0 |
| Moods swing between elation and depression | 0.74 | 0 | 0.37 | 0 | 0 | 0 | 0 |
| Physically attacks people | 0.69 | 0 | 0.41 | 0 | 0 | 0 | 0 |
| Screams or yells a lot | 0.65 | 0 | 0.58 | 0 | 0 | 0 | 0 |
| Very changeable behavior | 0.81 | 0 | 0.29 | 0 | 0 | 0 | 0 |
| Stubborn, sullen, or irritable | 0.59 | 0 | 0.50 | 0 | 0 | 0 | 0 |
| Sudden changes in mood or feelings | 0.70 | 0 | 0.52 | 0 | 0 | 0 | 0 |
| Temper tantrums or hot temper | 0.63 | 0 | 0.65 | 0 | 0 | 0 | 0 |
| Threatens to hurt people | 0.64 | 0 | 0.32 | 0 | 0 | 0 | 0 |
| Sulks a lot | 0.65 | 0 | 0.41 | 0 | 0 | 0 | 0 |
| Gets upset too easily | 0.66 | 0 | 0.51 | 0 | 0 | 0 | 0 |
| Is too impatient | 0.69 | 0 | 0.28 | 0 | 0 | 0 | 0 |
| Uses drugs (other than alcohol or nicotine) for nonmedical purposes | 0.48 | 0 | 0 | 0.57 | 0 | 0 | 0 |
| Breaks rules at work or elsewhere | 0.69 | 0 | 0 | 0.47 | 0 | 0 | 0 |
| Doesn’t seem to feel guilty after misbehaving | 0.61 | 0 | 0 | 0.22 | 0 | 0 | 0 |
| Hangs around people who get in trouble | 0.61 | 0 | 0 | 0.47 | 0 | 0 | 0 |
| Impulsive or acts without thinking | 0.65 | 0 | 0 | 0.32 | 0 | 0 | 0 |
| Lying or cheating | 0.70 | 0 | 0 | 0.38 | 0 | 0 | 0 |
| Irresponsible behavior | 0.77 | 0 | 0 | 0.37 | 0 | 0 | 0 |
| Drinks too much alcohol or gets drunk | 0.40 | 0 | 0 | 0.58 | 0 | 0 | 0 |
| Does things that may cause trouble with the law | 0.59 | 0 | 0 | 0.69 | 0 | 0 | 0 |
| Fails to pay his/her debts or meet other financial responsibilities | 0.66 | 0 | 0 | 0.58 | 0 | 0 | 0 |
| Has trouble managing money or credit cards | 0.64 | 0 | 0 | 0.53 | 0 | 0 | 0 |
| Has trouble keeping a job | 0.70 | 0 | 0 | 0.32 | 0 | 0 | 0 |
| Bragging, boasting | 0.40 | 0 | 0 | 0 | 0.55 | 0 | 0 |
| Demands a lot of attention | 0.62 | 0 | 0 | 0 | 0.39 | 0 | 0 |
| Showing off or clowning | 0.42 | 0 | 0 | 0 | 0.46 | 0 | 0 |
| Talks too much | 0.38 | 0 | 0 | 0 | 0.63 | 0 | 0 |
| Teases a lot | 0.44 | 0 | 0 | 0 | 0.43 | 0 | 0 |
| Is unusually loud | 0.69 | 0 | 0 | 0 | 0.43 | 0 | 0 |
| Complains of loneliness | 0.56 | 0 | 0 | 0 | 0 | 0.48 | 0 |
| Cries a lot | 0.55 | 0 | 0 | 0 | 0 | 0.45 | 0 |
| Fears he/she might think or do something bad | 0.44 | 0 | 0 | 0 | 0 | 0.53 | 0 |
| Feels or complains that no one loves him/her | 0.66 | 0 | 0 | 0 | 0 | 0.51 | 0 |
| Feels others are out to get him/her | 0.64 | 0 | 0 | 0 | 0 | 0.38 | 0 |
| Feels worthless or inferior | 0.71 | 0 | 0 | 0 | 0 | 0.56 | 0 |
| Nervous, highstrung, or tense | 0.67 | 0 | 0 | 0 | 0 | 0.47 | 0 |
| Lacks self-confidence | 0.65 | 0 | 0 | 0 | 0 | 0.53 | 0 |
| Too fearful or anxious | 0.63 | 0 | 0 | 0 | 0 | 0.58 | 0 |
| Feels to guilty | 0.55 | 0 | 0 | 0 | 0 | 0.52 | 0 |
| Self-conscious or easily embarrassed | 0.51 | 0 | 0 | 0 | 0 | 0.38 | 0 |
| Unhappy, sad, or depressed | 0.74 | 0 | 0 | 0 | 0 | 0.41 | 0 |
| Feels he/she can't succeed | 0.67 | 0 | 0 | 0 | 0 | 0.49 | 0 |
| Worries | 0.52 | 0 | 0 | 0 | 0 | 0.61 | 0 |
| Doesn’t get along with other people | 0.78 | 0 | 0 | 0 | 0 | 0 | 0.02 |
| Would rather be alone than with others | 0.39 | 0 | 0 | 0 | 0 | 0 | 0.77 |
| Not liked by others | 0.66 | 0 | 0 | 0 | 0 | 0 | 0.28 |
| Poor relations with opposite sex | 0.52 | 0 | 0 | 0 | 0 | 0 | 0.37 |
| Refuses to talk | 0.65 | 0 | 0 | 0 | 0 | 0 | 0.40 |
| Has trouble making or keeping friends | 0.59 | 0 | 0 | 0 | 0 | 0 | 0.61 |
| Secretive, keeps things to self | 0.52 | 0 | 0 | 0 | 0 | 0 | 0.58 |
| There is very little that he/she enjoys | 0.70 | 0 | 0 | 0 | 0 | 0 | 0.37 |
| Withdrawn, doesn't get involved with others | 0.48 | 0 | 0 | 0 | 0 | 0 | 0.83 |
| *Note.* The specific factors were assigned according to the instrument scales.  CATSS = Child and Adolescent Twin Study in Sweden.^5^ | | | | | | | |

**Supplementary Table 8B. Confirmatory bifactor factor analysis of 74 Adult Behavior Checklist symptoms in the CATSS sample (age 18), after fixing the loadings on the general factor in an inverted order.**

|  | **Factors** | | | | | | |
| --- | --- | --- | --- | --- | --- | --- | --- |
| **Item** | **General factor** | **Attention Problems** | **Aggressive Behavior** | **Rule-Breaking Behavior** | **Intrusive** | **Anxious/ Depressed** | **Withdrawn** |
| Is too forgetful | 0.72 | 0.15 | 0 | 0 | 0 | 0 | 0 |
| Can’t concentrate, can’t pay attention for long | 0.62 | 0.45 | 0 | 0 | 0 | 0 | 0 |
| Too dependent on others | 0.62 | 0.22 | 0 | 0 | 0 | 0 | 0 |
| Confused or seems to be in a fog | 0.52 | 0.46 | 0 | 0 | 0 | 0 | 0 |
| Daydreams or gets lost in his/her thoughts | 0.70 | 0.06 | 0 | 0 | 0 | 0 | 0 |
| Has trouble planning for the future | 0.56 | 0.55 | 0 | 0 | 0 | 0 | 0 |
| Fails to finish things he/she should do | 0.44 | 0.76 | 0 | 0 | 0 | 0 | 0 |
| Poor work performance | 0.44 | 0.69 | 0 | 0 | 0 | 0 | 0 |
| Has trouble setting priorities | 0.40 | 0.78 | 0 | 0 | 0 | 0 | 0 |
| Has trouble making decisions | 0.56 | 0.47 | 0 | 0 | 0 | 0 | 0 |
| Passive or lacks initiative | 0.43 | 0.63 | 0 | 0 | 0 | 0 | 0 |
| Stays away from school/job even when not sick and not on vacation | 0.65 | 0.28 | 0 | 0 | 0 | 0 | 0 |
| Underactive, slow moving, or lacks energy | 0.52 | 0.50 | 0 | 0 | 0 | 0 | 0 |
| Is disorganized | 0.66 | 0.32 | 0 | 0 | 0 | 0 | 0 |
| Tends to lose things | 0.73 | 0.03 | 0 | 0 | 0 | 0 | 0 |
| He/she is not good at details | 0.68 | 0.26 | 0 | 0 | 0 | 0 | 0 |
| Tends to be late for appointments | 0.70 | 0.16 | 0 | 0 | 0 | 0 | 0 |
| Argues a lot | 0.81 | 0 | -0.31 | 0 | 0 | 0 | 0 |
| Blames others for own problems | 0.69 | 0 | 0.23 | 0 | 0 | 0 | 0 |
| Cruelty, bullying, or meanness to others | 0.67 | 0 | 0.29 | 0 | 0 | 0 | 0 |
| Gets along badly with family | 0.61 | 0 | 0.48 | 0 | 0 | 0 | 0 |
| Gets in many fights | 0.42 | 0 | 0.48 | 0 | 0 | 0 | 0 |
| Moods swing between elation and depression | 0.40 | 0 | 0.75 | 0 | 0 | 0 | 0 |
| Physically attacks people | 0.55 | 0 | 0.59 | 0 | 0 | 0 | 0 |
| Screams or yells a lot | 0.64 | 0 | 0.59 | 0 | 0 | 0 | 0 |
| Very changeable behavior | 0.07 | 0 | 1.00 | 0 | 0 | 0 | 0 |
| Stubborn, sullen, or irritable | 0.66 | 0 | 0.45 | 0 | 0 | 0 | 0 |
| Sudden changes in mood or feelings | 0.48 | 0 | 0.76 | 0 | 0 | 0 | 0 |
| Temper tantrums or hot temper | 0.65 | 0 | 0.60 | 0 | 0 | 0 | 0 |
| Threatens to hurt people | 0.65 | 0 | 0.40 | 0 | 0 | 0 | 0 |
| Sulks a lot | 0.64 | 0 | 0.46 | 0 | 0 | 0 | 0 |
| Gets upset too easily | 0.61 | 0 | 0.57 | 0 | 0 | 0 | 0 |
| Is too impatient | 0.56 | 0 | 0.46 | 0 | 0 | 0 | 0 |
| Uses drugs (other than alcohol or nicotine) for nonmedical purposes | 0.70 | 0 | 0 | 0.32 | 0 | 0 | 0 |
| Breaks rules at work or elsewhere | 0.55 | 0 | 0 | 0.63 | 0 | 0 | 0 |
| Doesn’t seem to feel guilty after misbehaving | 0.66 | 0 | 0 | 0.26 | 0 | 0 | 0 |
| Hangs around people who get in trouble | 0.66 | 0 | 0 | 0.40 | 0 | 0 | 0 |
| Impulsive or acts without thinking | 0.64 | 0 | 0 | 0.37 | 0 | 0 | 0 |
| Lying or cheating | 0.51 | 0 | 0 | 0.63 | 0 | 0 | 0 |
| Irresponsible behavior | 0.39 | 0 | 0 | 0.84 | 0 | 0 | 0 |
| Drinks too much alcohol or gets drunk | 0.74 | 0 | 0 | 0.19 | 0 | 0 | 0 |
| Does things that may cause trouble with the law | 0.66 | 0 | 0 | 0.53 | 0 | 0 | 0 |
| Fails to pay his/her debts or meet other financial responsibilities | 0.61 | 0 | 0 | 0.62 | 0 | 0 | 0 |
| Has trouble managing money or credit cards | 0.65 | 0 | 0 | 0.53 | 0 | 0 | 0 |
| Has trouble keeping a job | 0.49 | 0 | 0 | 0.57 | 0 | 0 | 0 |
| Bragging, boasting | 0.74 | 0 | 0 | 0 | 0.10 | 0 | 0 |
| Demands a lot of attention | 0.66 | 0 | 0 | 0 | 0.49 | 0 | 0 |
| Showing off or clowning | 0.73 | 0 | 0 | 0 | -0.13 | 0 | 0 |
| Talks too much | 0.78 | 0 | 0 | 0 | 0.17 | 0 | 0 |
| Teases a lot | 0.72 | 0 | 0 | 0 | -0.19 | 0 | 0 |
| Is unusually loud | 0.52 | 0 | 0 | 0 | 0.40 | 0 | 0 |
| Complains of loneliness | 0.69 | 0 | 0 | 0 | 0 | 0.37 | 0 |
| Cries a lot | 0.69 | 0 | 0 | 0 | 0 | 0.33 | 0 |
| Fears he/she might think or do something bad | 0.72 | 0 | 0 | 0 | 0 | 0.21 | 0 |
| Feels or complains that no one loves him/her | 0.59 | 0 | 0 | 0 | 0 | 0.59 | 0 |
| Feels others are out to get him/her | 0.65 | 0 | 0 | 0 | 0 | 0.39 | 0 |
| Feels worthless or inferior | 0.48 | 0 | 0 | 0 | 0 | 0.83 | 0 |
| Nervous, highstrung, or tense | 0.59 | 0 | 0 | 0 | 0 | 0.55 | 0 |
| Lacks self-confidence | 0.63 | 0 | 0 | 0 | 0 | 0.57 | 0 |
| Too fearful or anxious | 0.65 | 0 | 0 | 0 | 0 | 0.53 | 0 |
| Feels to guilty | 0.69 | 0 | 0 | 0 | 0 | 0.36 | 0 |
| Self-conscious or easily embarrassed | 0.70 | 0 | 0 | 0 | 0 | 0.16 | 0 |
| Unhappy, sad, or depressed | 0.40 | 0 | 0 | 0 | 0 | 0.79 | 0 |
| Feels he/she can't succeed | 0.59 | 0 | 0 | 0 | 0 | 0.59 | 0 |
| Worries | 0.70 | 0 | 0 | 0 | 0 | 0.42 | 0 |
| Doesn’t get along with other people | 0.38 | 0 | 0 | 0 | 0 | 0 | 0.48 |
| Would rather be alone than with others | 0.77 | 0 | 0 | 0 | 0 | 0 | 0.39 |
| Not liked by others | 0.59 | 0 | 0 | 0 | 0 | 0 | 0.43 |
| Poor relations with opposite sex | 0.70 | 0 | 0 | 0 | 0 | 0 | 0.16 |
| Refuses to talk | 0.63 | 0 | 0 | 0 | 0 | 0 | 0.40 |
| Has trouble making or keeping friends | 0.67 | 0 | 0 | 0 | 0 | 0 | 0.53 |
| Secretive, keeps things to self | 0.69 | 0 | 0 | 0 | 0 | 0 | 0.38 |
| There is very little that he/she enjoys | 0.48 | 0 | 0 | 0 | 0 | 0 | 0.60 |
| Withdrawn, doesn't get involved with others | 0.71 | 0 | 0 | 0 | 0 | 0 | 0.57 |
| *Note.* The specific factors were assigned according to the instrument scales.  CATSS = Child and Adolescent Twin Study in Sweden.^5^ | | | | | | | |

**Supplementary Table 8C. 1-factor model of 74 Adult Behavior Checklist symptoms in the CATSS sample (age 18), before and after fixing the loadings on the general factor in an inverted order.**

| **Item** | **General factor** | **Inverted general factor** |
| --- | --- | --- |
| Is too forgetful | 0.52 | 0.74 |
| Can’t concentrate, can’t pay attention for long | 0.67 | 0.66 |
| Too dependent on others | 0.62 | 0.70 |
| Confused or seems to be in a fog | 0.69 | 0.63 |
| Daydreams or gets lost in his/her thoughts | 0.49 | 0.75 |
| Has trouble planning for the future | 0.68 | 0.64 |
| Fails to finish things he/she should do | 0.75 | 0.49 |
| Poor work performance | 0.73 | 0.59 |
| Has trouble setting priorities | 0.74 | 0.52 |
| Has trouble making decisions | 0.66 | 0.67 |
| Passive or lacks initiative | 0.70 | 0.62 |
| Stays away from school/job even when not sick and not on vacation | 0.61 | 0.70 |
| Underactive, slow moving, or lacks energy | 0.67 | 0.65 |
| Is disorganized | 0.62 | 0.70 |
| Tends to lose things | 0.48 | 0.75 |
| He/she is not good at details | 0.59 | 0.73 |
| Tends to be late for appointments | 0.53 | 0.74 |
| Argues a lot | 0.13 | 0.82 |
| Blames others for own problems | 0.58 | 0.73 |
| Cruelty, bullying, or meanness to others | 0.60 | 0.71 |
| Gets along badly with family | 0.69 | 0.64 |
| Gets in many fights | 0.70 | 0.62 |
| Moods swing between elation and depression | 0.76 | 0.45 |
| Physically attacks people | 0.73 | 0.58 |
| Screams or yells a lot | 0.74 | 0.53 |
| Very changeable behavior | 0.82 | 0.39 |
| Stubborn, sullen, or irritable | 0.65 | 0.68 |
| Sudden changes in mood or feelings | 0.77 | 0.43 |
| Temper tantrums or hot temper | 0.74 | 0.55 |
| Threatens to hurt people | 0.65 | 0.67 |
| Sulks a lot | 0.68 | 0.64 |
| Gets upset too easily | 0.72 | 0.59 |
| Is too impatient | 0.69 | 0.64 |
| Uses drugs (other than alcohol or nicotine) for nonmedical purposes | 0.55 | 0.74 |
| Breaks rules at work or elsewhere | 0.72 | 0.60 |
| Doesn’t seem to feel guilty after misbehaving | 0.59 | 0.72 |
| Hangs around people who get in trouble | 0.64 | 0.69 |
| Impulsive or acts without thinking | 0.64 | 0.68 |
| Lying or cheating | 0.71 | 0.61 |
| Irresponsible behavior | 0.78 | 0.42 |
| Drinks too much alcohol or gets drunk | 0.45 | 0.76 |
| Does things that may cause trouble with the law | 0.67 | 0.66 |
| Fails to pay his/her debts or meet other financial responsibilities | 0.71 | 0.60 |
| Has trouble managing money or credit cards | 0.67 | 0.66 |
| Has trouble keeping a job | 0.70 | 0.61 |
| Bragging, boasting | 0.40 | 0.79 |
| Demands a lot of attention | 0.60 | 0.72 |
| Showing off or clowning | 0.42 | 0.78 |
| Talks too much | 0.39 | 0.82 |
| Teases a lot | 0.43 | 0.77 |
| Is unusually loud | 0.68 | 0.65 |
| Complains of loneliness | 0.64 | 0.69 |
| Cries a lot | 0.62 | 0.70 |
| Fears he/she might think or do something bad | 0.53 | 0.74 |
| Feels or complains that no one loves him/her | 0.75 | 0.48 |
| Feels others are out to get him/her | 0.68 | 0.65 |
| Feels worthless or inferior | 0.82 | 0.13 |
| Nervous, highstrung, or tense | 0.74 | 0.53 |
| Lacks self-confidence | 0.74 | 0.53 |
| Too fearful or anxious | 0.73 | 0.57 |
| Feels to guilty | 0.64 | 0.68 |
| Self-conscious or easily embarrassed | 0.56 | 0.73 |
| Unhappy, sad, or depressed | 0.79 | 0.40 |
| Feels he/she can't succeed | 0.74 | 0.52 |
| Worries | 0.63 | 0.69 |
| Doesn’t get along with other people | 0.73 | 0.56 |
| Would rather be alone than with others | 0.52 | 0.74 |
| Not liked by others | 0.66 | 0.67 |
| Poor relations with opposite sex | 0.53 | 0.74 |
| Refuses to talk | 0.66 | 0.67 |
| Has trouble making or keeping friends | 0.65 | 0.68 |
| Secretive, keeps things to self | 0.57 | 0.73 |
| There is very little that he/she enjoys | 0.70 | 0.62 |
| Withdrawn, doesn't get involved with others | 0.61 | 0.71 |

**Supplementary Table 9A. Confirmatory bifactor factor analysis of 48 symptoms in the STAGE sample (age 20-45).**

| **Item** | **General factor** | **Int** | **Neuro** | **Sub** | **Imp** |
| --- | --- | --- | --- | --- | --- |
| Have you ever felt sad, blue or depressed for two weeks or more in a row? | 0.52 | 0.27 | 0 | 0 | 0 |
| Have you ever had a period lasting one month or longer when most of the time you felt worried and anxious? | 0.53 | 0.27 | 0 | 0 | 0 |
| Excessive cleaning: hand washing, baths, showers, toothbrushing etc? | 0.48 | 0.65 | 0 | 0 | 0 |
| Other special measures to avoid dirt, germs or poisons? | 0.45 | 0.67 | 0 | 0 | 0 |
| Excessive checking: electric switches, gas taps, locks, doors, the oven? | 0.37 | 0.63 | 0 | 0 | 0 |
| Repeating the same simple activity many times in a row for no reason, e.g. repeatedly standing up or sitting down or going backwards and forwards through a doorway? | 0.44 | 0.74 | 0 | 0 | 0 |
| Touching things or people in particular ways? | 0.46 | 0.70 | 0 | 0 | 0 |
| Arranging things so they are just so, or exactly symmetrical? | 0.49 | 0.62 | 0 | 0 | 0 |
| Counting to particular lucky numbers or avoiding unlucky numbers? | 0.37 | 0.64 | 0 | 0 | 0 |
| Do you have or have you ever had depression? | 0.56 | 0.22 | 0 | 0 | 0 |
| Do you have or have you ever had panic attacks? | 0.54 | 0.23 | 0 | 0 | 0 |
| Do you have or have you ever had phobia? | 0.47 | 0.31 | 0 | 0 | 0 |
| Do you have difficulties expressing emotions and reactions with facial gestures, pronunciation, or body language? | 0.30 | 0 | 0.64 | 0 | 0 |
| Have you difficulties to get and keep friends? | 0.39 | 0 | 0.41 | 0 | 0 |
| Are you disinterested in sharing joy, interests, and activities with others? | 0.24 | 0 | 0.26 | 0 | 0 |
| Can you only be with other people on your terms? | 0.53 | 0 | 0.23 | 0 | 0 |
| Were your language development delayed? | 0.21 | 0 | 0.27 | 0 | 0 |
| Do you have difficulties participating in discussions with others? | 0.44 | 0 | 0.65 | 0 | 0 |
| Do you have difficulty imitating other people or to play charades? | 0.18 | 0 | 0.51 | 0 | 0 |
| Do you get absorbed by your interests in such a way as being repetitive or too intense? | 0.48 | 0 | 0.10 | 0 | 0 |
| Do you get absorbed by routines in such a way as to produce problems for yourself or for others? | 0.57 | 0 | 0.14 | 0 | 0 |
| Do you get absorbed by details? | 0.57 | 0 | 0.10 | 0 | 0 |
| Thinking about the whole of your life, have you ever had motor tics involving any of the following types of repeated movement? Excessive blinking of eyes | 0.27 | 0 | -0.15 | 0 | 0 |
| Thinking about the whole of your life, have you ever had vocal tics involving any of the following types of repeated sounds? | 0.29 | 0 | -0.03 | 0 | 0 |
| Do you often fail to pay close attention to details or make careless mistakes when you write, or other activities? | 0.48 | 0 | 0.25 | 0 | 0 |
| Do you often have difficulty sustaining attention in tasks or activities? | 0.67 | 0 | 0.35 | 0 | 0 |
| Do you often seem not to listen when spoken to directly? | 0.57 | 0 | 0.23 | 0 | 0 |
| Do you often fail to follow instructions and to finish tasks? | 0.64 | 0 | 0.43 | 0 | 0 |
| Do you often have difficulty organising tasks and activities? | 0.55 | 0 | 0.57 | 0 | 0 |
| Do you often avoid tasks that require sustained mental effort? | 0.57 | 0 | 0.39 | 0 | 0 |
| Do you often lose things? | 0.46 | 0 | 0.17 | 0 | 0 |
| Are you often easily distracted or disturbed? | 0.64 | 0 | 0.24 | 0 | 0 |
| Are you often forgetful in daily activities? | 0.51 | 0 | 0.24 | 0 | 0 |
| Have you ever thought that you should limit your alcohol consumption? | 0.37 | 0 | 0 | 0.79 | 0 |
| Have other people irritated you by criticizing your way of drinking? | 0.41 | 0 | 0 | 0.76 | 0 |
| Have you ever felt bad or have you had feelings of guilt due to your way of drinking? | 0.40 | 0 | 0 | 0.75 | 0 |
| Have you ever been drinking the first thing in the morning to calm your nerves or to cure a hangover | 0.35 | 0 | 0 | 0.59 | 0 |
| Have you ever tried Marijuana | 0.26 | 0 | 0 | 0.53 | 0 |
| Have you ever tried hash | 0.32 | 0 | 0 | 0.54 | 0 |
| Do you have difficulties holding your hands and feet still or can you not stay seated? | 0.53 | 0 | 0 | 0 | 0.31 |
| Do you get up and move about in situations when you are supposed to remain seated? | 0.58 | 0 | 0 | 0 | 0.20 |
| Are you restless? | 0.57 | 0 | 0 | 0 | 0.42 |
| Do you have difficulty doing calm leisure pursuit? | 0.48 | 0 | 0 | 0 | 0.46 |
| Does it often feel like you are ”on the go”? | 0.49 | 0 | 0 | 0 | 0.57 |
| Do you often talk excessively? | 0.36 | 0 | 0 | 0 | 0.61 |
| Do you often blurt out answers before the question has been completed? | 0.46 | 0 | 0 | 0 | 0.57 |
| Do you have difficulty awaiting turns? | 0.51 | 0 | 0 | 0 | 0.56 |
| Do you often interrupt or intrude on others? | 0.43 | 0 | 0 | 0 | 0.50 |

***Note.* The specific factors were assigned according to the (exploratory) loading pattern identified in a previous study.^6^**

**Int = Specific internalizing factor. Neuro = Specific neurodevelopmental factor. Sub = Specific substance misuse factor. Imp = Specific impulsivity factor.**

**Supplementary Table 9B. Confirmatory bifactor factor analysis of 48 symptoms in the STAGE sample (age 20-45), after fixing the loadings on the general factor in an inverted order.**

| **Item** | **General factor** | **Int** | **Neuro** | **Sub** | **Imp** |
| --- | --- | --- | --- | --- | --- |
| Have you ever felt sad, blue or depressed for two weeks or more in a row? | 0.41 | 0.69 | 0 | 0 | 0 |
| Have you ever had a period lasting one month or longer when most of the time you felt worried and anxious? | 0.40 | 0.69 | 0 | 0 | 0 |
| Excessive cleaning: hand washing, baths, showers, toothbrushing etc? | 0.46 | 0.42 | 0 | 0 | 0 |
| Other special measures to avoid dirt, germs or poisons? | 0.49 | 0.38 | 0 | 0 | 0 |
| Excessive checking: electric switches, gas taps, locks, doors, the oven? | 0.54 | 0.23 | 0 | 0 | 0 |
| Repeating the same simple activity many times in a row for no reason, e.g. repeatedly standing up or sitting down or going backwards and forwards through a doorway? | 0.49 | 0.37 | 0 | 0 | 0 |
| Touching things or people in particular ways? | 0.48 | 0.38 | 0 | 0 | 0 |
| Arranging things so they are just so, or exactly symmetrical? | 0.45 | 0.37 | 0 | 0 | 0 |
| Counting to particular lucky numbers or avoiding unlucky numbers? | 0.53 | 0.21 | 0 | 0 | 0 |
| Do you have or have you ever had depression? | 0.36 | 0.75 | 0 | 0 | 0 |
| Do you have or have you ever had panic attacks? | 0.37 | 0.67 | 0 | 0 | 0 |
| Do you have or have you ever had phobia? | 0.48 | 0.40 | 0 | 0 | 0 |
| Do you have difficulties expressing emotions and reactions with facial gestures, pronunciation, or body language? | 0.57 | 0 | 0.15 | 0 | 0 |
| Have you difficulties to get and keep friends? | 0.53 | 0 | 0.22 | 0 | 0 |
| Are you disinterested in sharing joy, interests, and activities with others? | 0.64 | 0 | 0.01 | 0 | 0 |
| Can you only be with other people on your terms? | 0.37 | 0 | 0.43 | 0 | 0 |
| Were your language development delayed? | 0.64 | 0 | 0.01 | 0 | 0 |
| Do you have difficulties participating in discussions with others? | 0.51 | 0 | 0.33 | 0 | 0 |
| Do you have difficulty imitating other people or to play charades? | 0.67 | 0 | 0.00 | 0 | 0 |
| Do you get absorbed by your interests in such a way as being repetitive or too intense? | 0.46 | 0 | 0.33 | 0 | 0 |
| Do you get absorbed by routines in such a way as to produce problems for yourself or for others? | 0.29 | 0 | 0.43 | 0 | 0 |
| Do you get absorbed by details? | 0.35 | 0 | 0.45 | 0 | 0 |
| Thinking about the whole of your life, have you ever had motor tics involving any of the following types of repeated movement? Excessive blinking of eyes | 0.57 | 0 | -0.03 | 0 | 0 |
| Thinking about the whole of your life, have you ever had vocal tics involving any of the following types of repeated sounds? | 0.57 | 0 | 0.01 | 0 | 0 |
| Do you often fail to pay close attention to details or make careless mistakes when you write, or other activities? | 0.47 | 0 | 0.46 | 0 | 0 |
| Do you often have difficulty sustaining attention in tasks or activities? | 0.18 | 0 | 0.85 | 0 | 0 |
| Do you often seem not to listen when spoken to directly? | 0.27 | 0 | 0.56 | 0 | 0 |
| Do you often fail to follow instructions and to finish tasks? | 0.21 | 0 | 0.76 | 0 | 0 |
| Do you often have difficulty organising tasks and activities? | 0.37 | 0 | 0.61 | 0 | 0 |
| Do you often avoid tasks that require sustained mental effort? | 0.30 | 0 | 0.60 | 0 | 0 |
| Do you often lose things? | 0.48 | 0 | 0.43 | 0 | 0 |
| Are you often easily distracted or disturbed? | 0.24 | 0 | 0.72 | 0 | 0 |
| Are you often forgetful in daily activities? | 0.44 | 0 | 0.52 | 0 | 0 |
| Have you ever thought that you should limit your alcohol consumption? | 0.55 | 0 | 0 | 0.70 | 0 |
| Have other people irritated you by criticizing your way of drinking? | 0.52 | 0 | 0 | 0.75 | 0 |
| Have you ever felt bad or have you had feelings of guilt due to your way of drinking? | 0.53 | 0 | 0 | 0.75 | 0 |
| Have you ever been drinking the first thing in the morning to calm your nerves or to cure a hangover | 0.57 | 0 | 0 | 0.43 | 0 |
| Have you ever tried Marijuana | 0.58 | 0 | 0 | 0.19 | 0 |
| Have you ever tried hash | 0.57 | 0 | 0 | 0.24 | 0 |
| Do you have difficulties holding your hands and feet still or can you not stay seated? | 0.39 | 0 | 0 | 0 | 0.54 |
| Do you get up and move about in situations when you are supposed to remain seated? | 0.26 | 0 | 0 | 0 | 0.57 |
| Are you restless? | 0.32 | 0 | 0 | 0 | 0.70 |
| Do you have difficulty doing calm leisure pursuit? | 0.46 | 0 | 0 | 0 | 0.58 |
| Does it often feel like you are ”on the go”? | 0.44 | 0 | 0 | 0 | 0.66 |
| Do you often talk excessively? | 0.56 | 0 | 0 | 0 | 0.37 |
| Do you often blurt out answers before the question has been completed? | 0.48 | 0 | 0 | 0 | 0.47 |
| Do you have difficulty awaiting turns? | 0.43 | 0 | 0 | 0 | 0.56 |
| Do you often interrupt or intrude on others? | 0.51 | 0 | 0 | 0 | 0.38 |

***Note.* The specific factors were assigned according to the (exploratory) loading pattern identified in a previous study.^6^**

**Int = Specific internalizing factor. Neuro = Specific neurodevelopmental factor. Sub = Specific substance misuse factor. Imp = Specific impulsivity factor.**

**Supplementary Table 9C. 1- factor models of 48 symptoms in the STAGE sample (age 20-45) , before and after fixing the loadings on the general factor in an inverted order.**

| **Item** | **General factor** | **Inverted general factor** |
| --- | --- | --- |
| Have you ever felt sad, blue or depressed for two weeks or more in a row? | 0.52 | 0.41 |
| Have you ever had a period lasting one month or longer when most of the time you felt worried and anxious? | 0.53 | 0.40 |
| Excessive cleaning: hand washing, baths, showers, toothbrushing etc? | 0.48 | 0.46 |
| Other special measures to avoid dirt, germs or poisons? | 0.45 | 0.49 |
| Excessive checking: electric switches, gas taps, locks, doors, the oven? | 0.37 | 0.54 |
| Repeating the same simple activity many times in a row for no reason, e.g. repeatedly standing up or sitting down or going backwards and forwards through a doorway? | 0.44 | 0.49 |
| Touching things or people in particular ways? | 0.46 | 0.48 |
| Arranging things so they are just so, or exactly symmetrical? | 0.49 | 0.45 |
| Counting to particular lucky numbers or avoiding unlucky numbers? | 0.37 | 0.53 |
| Do you have or have you ever had depression? | 0.56 | 0.36 |
| Do you have or have you ever had panic attacks? | 0.54 | 0.37 |
| Do you have or have you ever had phobia? | 0.47 | 0.48 |
| Do you have difficulties expressing emotions and reactions with facial gestures, pronunciation, or body language? | 0.30 | 0.57 |
| Have you difficulties to get and keep friends? | 0.39 | 0.53 |
| Are you disinterested in sharing joy, interests, and activities with others? | 0.24 | 0.64 |
| Can you only be with other people on your terms? | 0.53 | 0.37 |
| Were your language development delayed? | 0.21 | 0.64 |
| Do you have difficulties participating in discussions with others? | 0.44 | 0.51 |
| Do you have difficulty imitating other people or to play charades? | 0.18 | 0.67 |
| Do you get absorbed by your interests in such a way as being repetitive or too intense? | 0.48 | 0.46 |
| Do you get absorbed by routines in such a way as to produce problems for yourself or for others? | 0.57 | 0.29 |
| Do you get absorbed by details? | 0.57 | 0.35 |
| Thinking about the whole of your life, have you ever had motor tics involving any of the following types of repeated movement? Excessive blinking of eyes | 0.27 | 0.57 |
| Thinking about the whole of your life, have you ever had vocal tics involving any of the following types of repeated sounds? | 0.29 | 0.57 |
| Do you often fail to pay close attention to details or make careless mistakes when you write, or other activities? | 0.48 | 0.47 |
| Do you often have difficulty sustaining attention in tasks or activities? | 0.67 | 0.18 |
| Do you often seem not to listen when spoken to directly? | 0.57 | 0.27 |
| Do you often fail to follow instructions and to finish tasks? | 0.64 | 0.21 |
| Do you often have difficulty organising tasks and activities? | 0.55 | 0.37 |
| Do you often avoid tasks that require sustained mental effort? | 0.57 | 0.30 |
| Do you often lose things? | 0.46 | 0.48 |
| Are you often easily distracted or disturbed? | 0.64 | 0.24 |
| Are you often forgetful in daily activities? | 0.51 | 0.44 |
| Have you ever thought that you should limit your alcohol consumption? | 0.37 | 0.55 |
| Have other people irritated you by criticizing your way of drinking? | 0.41 | 0.52 |
| Have you ever felt bad or have you had feelings of guilt due to your way of drinking? | 0.40 | 0.53 |
| Have you ever been drinking the first thing in the morning to calm your nerves or to cure a hangover | 0.35 | 0.57 |
| Have you ever tried Marijuana | 0.26 | 0.58 |
| Have you ever tried hash | 0.32 | 0.57 |
| Do you have difficulties holding your hands and feet still or can you not stay seated? | 0.53 | 0.39 |
| Do you get up and move about in situations when you are supposed to remain seated? | 0.58 | 0.26 |
| Are you restless? | 0.57 | 0.32 |
| Do you have difficulty doing calm leisure pursuit? | 0.48 | 0.46 |
| Does it often feel like you are ”on the go”? | 0.49 | 0.44 |
| Do you often talk excessively? | 0.36 | 0.56 |
| Do you often blurt out answers before the question has been completed? | 0.46 | 0.48 |
| Do you have difficulty awaiting turns? | 0.51 | 0.43 |
| Do you often interrupt or intrude on others? | 0.43 | 0.51 |

*Note.* The STAGE sample is described elsewhere.^6^

**Supplementary Table 10. Power simulation of hierarchical factor analytic models regressed on a covariate.**

|  | Power of Q_SNP_ comparison to detect regression of general factor (p) on covariate^1^ | | Power to detect regression of general factor (p) on covariate |
| --- | --- | --- | --- |
| Simulated population model:  Regression of general (p) and specific factors (S) on covariate^2^ | Sample model: Bifactor model Q_SNP_ comparison | Sample model:  Higher-order model Q_SNP_ comparison | Sample model:  Covariate -> bifactor model (p, S1, S2, S3, S4) |
| Covariate -> p | 96% | 95% | 100% |
| Covariate -> p, S1 | 13% | 4% | 100% |
| Covariate -> p, S2 | 21% | 13% | 100% |
| Covariate -> p, S3 | 10% | 5% | 100% |
| Covariate -> p, S4 | 39% | 10% | 100% |
| Covariate -> p, S1, S2 | 3% | 1% | 100% |
| Covariate -> p, S1, S3 | 0% | 1% | 100% |
| Covariate -> p, S1, S4 | 7% | 7% | 100% |
| Covariate -> p, S2, S3 | 1% | 1% | 100% |
| Covariate -> p, S2, S4 | 13% | 9% | 100% |
| Covariate -> p, S3, S4 | 6% | 10% | 100% |
| Covariate -> p, S1, S2, S3 | 0% | 1% | 100% |
| Covariate -> p, S1, S2, S3, S4 | 0% | -^3^ | 100% |

*Note.* The measurement models were always matched for the simulated population and the sample (e.g., when the simulated population measurement model corresponded to a bifactor model, so did the sample model). A total of 100 samples (*N* = 10,000) were drawn from the population, and the population regression beta on p equaled .08, and 0.04 for the specific factor(s), for all simulations. See text for further simulation and Q_SNP_ comparison details. p means general factor; S1 (specific factor 1) captured primarily obsessive-compulsive disorder and anorexia; S2 captured schizophrenia and bipolar disorder; S3 captured primarily ADHD, autism, and post-traumatic stress disorder; and S4 captured primarily anxiety and depression. The full set of factor loadings are available in the original publication.^7^

^1^ For row 1, the percentage captures how often the Q_SNP_ comparison correctly identified the simulated association between the covariate and the general factor (i.e., its statistical power). For the remaining rows, the percentage represents how often the Q_SNP_ comparison rejected the simpler model (where the covariate was only associated with the general factor) in favor of the more complex model (where the covariate was primarily associated with the specific factors). For these rows, given that the covariate was simulated to be associated with both the general and one (or more) specific factors, a low percentage indicates that the Q_SNP_ comparison is biased toward incorrectly rejecting the utility of the general factor. See text for further details on the Q_SNP_ comparison approach.

^2^ This column displays the simulated population. For instance, “Covariate -> p, S1” means that the general factor and specific factor 1 were associated with the covariate in the simulated population (and that the covariate was not associated with specific factors 2-4).

^3^ This model cannot be estimated because it lacks one degree of freedom.^8^

**References**

1. Fried EI, Greene AL, Eaton NR. The p factor is the sum of its parts, for now. *World Psychiatry*. Feb 2021;20(1):69-70. doi:10.1002/wps.20814

2. Pettersson E, Larsson H, D'Onofrio BM, Bolte S, Lichtenstein P. The general factor of psychopathology: a comparison with the general factor of intelligence with respect to magnitude and predictive validity. *World Psychiatry*. Jun 2020;19(2):206-213. doi:10.1002/wps.20763

3. Levin-Aspenson HF, Watson D, Clark LA, Zimmerman M. What Is the General Factor of Psychopathology? Consistency of the p Factor Across Samples. *Assessment*. Jun 2021;28(4):1035-1049. doi:10.1177/1073191120954921

4. Pettersson E, Lichtenstein P, Larsson H, D'Onofrio BM, Lahey BB, Latvala A. Associations of Resting Heart Rate and Intelligence With General and Specific Psychopathology: A Prospective Population Study of 899,398 Swedish Men. *Clinical Psychological Science*. Mar 15 2021;doi:10.1177/2167702620961081

5. Anckarsater H, Lundstrom S, Kollberg L, et al. The Child and Adolescent Twin Study in Sweden (CATSS). *Twin Research and Human Genetics*. Dec 2011;14(6):495-508. doi:DOI 10.1375/twin.14.6.495

6. Pettersson E, Larsson H, D'Onofrio BM, Lichtenstein P. Associations Between General and Specific Psychopathology Factors and 10-Year Clinically Relevant Outcomes in Adult Swedish Twins and Siblings. *Jama Psychiat*. Jul 1 2023;80(7):728-737. doi:10.1001/jamapsychiatry.2023.1162

7. Grotzinger AD, Mallard TT, Akingbuwa WA, et al. Genetic architecture of 11 major psychiatric disorders at biobehavioral, functional genomic and molecular genetic levels of analysis. *Nat Genet*. May 2022;54(5):548-559. doi:10.1038/s41588-022-01057-4

8. Moore TM, Kaczkurkin AN, Durham EL, et al. Criterion validity and relationships between alternative hierarchical dimensional models of general and specific psychopathology. *J Abnorm Psychol*. Oct 2020;129(7):677-688. doi:10.1037/abn0000601
